# N-acetylcysteine and raloxifene boost photodynamic therapy against cutaneous squamous cell carcinoma by decreasing TGFβ1 secreted by cancer-associated fibroblasts

**DOI:** 10.7150/ijbs.106642

**Published:** 2025-04-28

**Authors:** María Gallego-Rentero, Luisa María Botella, Marta Mascaraque, Jimena Nicolás-Morala, Virginia Albiñana, Edgar Abarca-Lachen, Yolanda Gilaberte, Salvador González, Ángeles Juarranz, Elisa Carrasco

**Affiliations:** 1 Departamento de Biología, Universidad Autónoma de Madrid, 28049, Madrid, Spain.; 2 Instituto Ramón y Cajal de Investigación Sanitaria, IRYCIS, 28034, Madrid, Spain.; 3 Centro de Investigaciones Biológicas Margarita Salas | CSIC, 28040, Madrid, Spain.; 4 La Paz Hospital, 28029, Madrid, Spain.; 5 Facultad de Ciencias de la Salud, Universidad de San Jorge, 22300, Barbastro, Spain.; 6 Servicio de dermatología, Miguel Servet Hospital, 50009, Zaragoza, Spain.; 7 Department of Medicine and Medical Specialties, Alcalá de Henares University, 28805, Madrid, Spain; 8 Centro de Biología Molecular Severo Ochoa (CBM); Instituto Universitario de Biología Molecular-IUBM (Universidad Autónoma de Madrid), 28049, Madrid, Spain.

**Keywords:** cutaneous squamous cell carcinoma, photodynamic therapy, TGFβ1, cell cycle arrest, endoglin, N-acetylcysteine, raloxifene

## Abstract

Cutaneous squamous cell carcinoma (cSCC) is a highly prevalent skin cancer. While surgery remains the gold standard treatment, non-invasive methods like photodynamic therapy (PDT) stand out for their high efficacy and minimal cosmetic impact. However, resistance to PDT is still a challenge. Numerous cellular processes involved in cancer biology and therapy resistance are regulated by the TGFβ1/SMAD pathway. Using *in vitro* bidimensional and tridimensional cultures of cSCC cell lines, we studied the development of resistance to PDT in response to TGFβ1 secreted by cancer associated fibroblasts. Our results highlight the TGFβ1 co-receptor endoglin as a key molecular player in the process. Importantly, targeting endoglin expression with N-acetylcysteine (NAC) or raloxifene significantly reduced TGFβ1 levels and effectively prevented resistance. In addition, the combination of PDT with NAC resulted in an improved therapeutic outcome *in vivo* in SKH-1 mice with cSCC photogenerated by chronic exposition to ultraviolet light. In conclusion, the combination of PDT with NAC or raloxifene enhances PDT efficacy by mitigating resistance mechanisms, which can open new avenues for the treatment of cSCC.

## 1. Introduction

Cutaneous squamous cell carcinoma (cSCC) is the most common metastatic skin cancer with increasing incidence worldwide. Among the treatments available, surgery is still the gold standard. However, cosmetic limitations strongly motivate the use of non-invasive procedures, such as photodynamic therapy (PDT) 1,2. PDT is a treatment modality that combines three elements to induce cell death in targeted tissues: a photosensitizing agent, light of a specific wavelength, and molecular oxygen. Generally used for the treatment of tumoral malignancies, PDT has gained prominence as a minimally invasive therapy that efficiently eliminates skin cancer lesions 3. One of the primary advantages of PDT is its selectivity, as the cytotoxic effects are confined to the irradiated area, sparing surrounding healthy tissues. In addition, PDT can be repeated at the same site if necessary and has minimal systemic side effects compared to conventional therapies such as chemotherapy and radiation. Despite its effectiveness, some tumor cells can develop resistance to therapy. Therefore, understanding the mechanisms of resistance can help to improve treatment outcomes 4,5. In particular, SCCs exhibit high rates of tumor recurrence following anti-cancer therapy and recurrent tumors are likely more aggressive and resistant to treatments. Previous studies have already addressed this issue describing Transforming Growth Factor Beta (TGFβ) activation as one of the many possible culprits 6,7.

TGFβ is a cytokine with three functionally different isoforms: TGFβ1, TGFβ2, and TGFβ3. Specifically, TGFβ1 is the most abundant isoform and constitutes a powerful cytokine that plays a critical role in regulating different cellular processes. It is secreted by various cell types (including immune cells, epithelial cells and fibroblasts) in an inactive form that needs activation. Once the latent TGFβ1 ligands are processed and activated, they bind to their receptors, TβRII and TβRI. TβRI then continues the signaling process by phosphorylating the downstream intracellular effectors SMAD2 and SMAD3. Upon binding to SMAD4, the P-SMAD2/3-SMAD4 complexes are imported into the nucleus and bind specific DNA sequence motifs known as SMAD-binding elements to activate TGFβ1-responsive target genes. This signaling cascade impacts diverse cellular processes such as cell proliferation. In cancer, TGFβ1 exhibits a dual role. While acting as a tumor suppressor in early stages by inhibiting cell proliferation and inducing apoptosis, in later stages it can promote tumor progression by enhancing epithelial-to-mesenchymal transition (EMT), angiogenesis, and immune evasion 8,9. In the context of its tumor suppressor role, TGFβ1 signaling is able to induce the expression of P21, a critical protein that regulates the cell cycle by inhibiting cyclin-dependent kinases. This action leads to cell cycle arrest, allowing quiescent cancer cells to escape the effect of anti-tumoral drugs 10-12. This intricate network highlights the dual role of TGFβ1 in tumor suppression and therapy resistance, emphasizing its potential as a therapeutic target in cancer treatment strategies, including PDT.

The TGFβ1 signaling pathway can be modulated by its co-receptor endoglin (also known as CD105) by enhancing or inhibiting the binding of the cytokine to its primary receptors, depending on the cellular context. Endoglin is a homodimeric membrane glycoprotein formed by two 90 kDa subunits. It has a large extracellular domain, a single transmembrane region and a short cytoplasmic tail, and it is expressed in different cell types including fibroblasts 13. Membrane endoglin can compete with TGFβ1 receptors for ligand binding, reducing the ligand availability for TβRII and TβRI. Moreover, it can influence the formation and function of TGFβ1 receptor complexes, altering the downstream signaling dynamics. In addition, membrane endoglin overexpression can lead to its cleavage generating a soluble active form that consists of the extracellular portion of the membrane protein. Soluble endoglin can also bind TGFβ1, thereby acting as a decoy receptor and inhibiting TGFβ1 signaling 14-16. Thus, the role of endoglin highlights the complexity of TGFβ1 signaling regulation and the importance of understanding these dynamics for potential therapeutic interventions.

In this work we propose the combination of PDT with the approved drugs raloxifene and N-acetylcysteine (NAC) as a strategy to overcome PDT resistance in cSCC cells. Raloxifene, known for its role as a selective estrogen receptor modulator used in osteoporosis treatment, influences TGFβ1 signaling pathways 17. It can modulate the expression of TGFβ1 and its receptors, including the synthesis of endoglin, affecting critical cellular processes such as proliferation, differentiation, and apoptosis. NAC acts as an antioxidant and precursor of glutathione, commonly used as a mucolytic 17. NAC has been described to inhibit TGFβ1 signaling by reducing the activation of SMAD proteins, which are key downstream effectors of TGFβ1. NAC influence extends to the expression and activity of endoglin, impacting TGFβ1 signaling 18. These effects of raloxifene and NAC on TGFβ1 signaling and endoglin expression are particularly promising to favor cancer therapy. The potential of these compounds to target complex diseases linked to TGFβ1 and endoglin motivates their use in combination with PDT, with the aim of enhancing treatment efficacy by mitigating tumor resistance 17-19. Bearing this in mind, in this work we propose the repositioning of these two well-known drugs as co-adjuvants of PDT for the treatment of cSCC.

## 2. Methods and materials

### 2.1. Cell culture

A431 is a cell line obtained from a poorly differentiated cSCC of an 85-year-old female and purchased from the American Type Culture Collection (ATCC, Manassas, VA, US). SCC13 cell line corresponds to a moderately differentiated facial SCC of a 56-year-old female kindly provided by Dr. Quintanilla (Instituto de Investigaciones Biomédicas Sols-Morreale, Madrid, Spain).

Cancer associated fibroblast (CAFs) primary cultures were obtained from biopsies of adult patients with *in situ* or invasive cSCC (T165A and T205A, respectively), following the procedure indicated in previously published studies 23. Briefly, biopsies were chopped and incubated with 5 ml dispase II (Sigma, St. Louis, MO, USA) overnight at 37ºC, followed by centrifugation and pellet transference onto a flask with culture medium. Control fibroblasts (Fb) were purchased from Innoprot (Innovative Technologies in Biological Systems, Derio, Spain) and derive from the dermis of a healthy adult.

cSCC cell lines and fibroblasts were cultured in complete medium, composed of Dulbecco's Modified Eagle Medium (DMEM) supplemented with 10% (v/v) fetal bovine serum (FBS), 1% Penicillin G (100 U/ml) and streptomycin (100 µg/ml), all from Fisher Scientific Inc. (Rockford, IL, USA). Passages were carried out using 0.05% or 0.25% Trypsin (w/v) (for fibroblasts or keratinocyte cell lines, respectively), containing 1 mM EDTA (Fisher Scientific Inc., Rockford, IL, USA). Cells were maintained in an incubator (Heraeus HERAcell, Thermo Fisher Scientific Waltham, MA, USA) at 37 ᵒC, 5% humidity, 5% CO_2_. All experiments were carried out with cells doublings between 3 and 9.

Spheroid three-dimensional cultures were prepared using A431 cells seeded on multi-well plates pre‑coated overnight at room temperature with 1.2% poly-HEMA (2-hydroxyethyl methacrylate, Sigma-Aldrich) to prevent cell adhesion, and maintained in a specific medium made of 1:1 DMEM:F12 (F-12 Nutrient mixture, Ham, Gibco, Thermo Fisher Scientific Inc., Rockford, IL, USA), 2% B27 serum free supplement (Gibco), 20 ng/ml of EGF (Sigma-Aldrich/Merck), 0.4% BSA and 4 µg/ml of insulin (Gibco). Cells were plated at a density of 40,000 cells/ml and the spheroids were formed over a period of 6 days.

### 2.2. Cell treatment with conditioned medium from fibroblasts

Conditioned medium (CM) for cell treatments was obtained from fibroblasts (both CAFs and Fb) cultured in 75 cm^2^ flasks. When 80% confluence was reached, fibroblasts' medium was replaced with DMEM supplemented with 1% FBS and 1% antibiotics. At this time point, NAC or raloxifene were added to the medium when appropriate. NAC stock (100 mM, pH 7.4, Sigma-Aldrich, St. Louis, MO, USA) was prepared in PBS. Raloxifene stock (54 mM in DMSO, Sigma-Aldrich, St. Louis, MO, USA) was kindly provided by Dr. Botella (Centro de Investigaciones Biológicas, CSIC, Madrid, Spain). After a 24 h incubation period, the CM was collected and freshly used for cell treatment or stored at -80 ºC until used for ELISA measurements. cSCC cells were treated with CM from either CAFs or Fb. Treatments were performed 24 h after seeding the cells and maintained until the time point of evaluation.

For PDT resistance studies, either A431 and SCC13 bidimensional cultures that reached 30-40% confluence 24 h after cell seeding, or A431 spheroids formed over 6 days, were incubated for the next 48 h with CM alone or CM previously collected in the presence of either NAC or raloxifene.

### 2.3. Cell treatment with photodynamic therapy

A 10 mM stock of methyl-aminolevulinate (MAL) (Sigma-Aldrich, St. Louis, MO, USA) was prepared in deionized sterile water. Then, cSCC cells at 60-70% confluence or completely formed spheroids were incubated with 0.5 mM MAL in DMEM without FBS for 5 h and subsequently irradiated with red light doses ranging between 0.6 and 12.2 J·cm^-2^. A red-light emitting diode source (WP7143 SURC/E Kingsbright, Angels, CA, USA) with an irradiation intensity of 6.2 mW·cm^-2^ and an emission peak at λ = 634 ± 20 nm was used. These treatment conditions were selected as capable of inducing 50% of cell death in SCC13 and A431 according to previous works by our research group 23. After PDT, the medium was replaced with fresh medium with the corresponding composition.

### 2.4. Assessment of cell toxicity and migratory capacity

The toxicity of MAL-PDT in cells was assessed 24 h after the phototreatments. To this end, the 3-(4,5-dimethylthiazol-2-yl)-2,5-diphenyltetrazolium bromide (MTT) assay was used to assess the toxicity of MAL-PDT in bidimensional cultures 24 h after MAL-PDT administration. To this end, a 1 mg/ml MTT (Sigma-Aldrich, St. Louis, MO, USA) stock was prepared in PBS and diluted at 50 µg/ml in complete medium. Culture media were replaced with the MTT solution and incubated for 3 h at 37 ᵒC. After removing the solution, formazan crystals were dissolved in DMSO (Panreac, Barcelona, Spain) and the absorbance at 542 nm was measured using a SpectraFluor, Tecan (Zürich, Switzerland) plate reader to estimate the metabolic activity of the cultures. The results were normalized and presented as relative absorbance (percentage of the control). The toxicity of MAL-PDT in spheroids was determined by propidium iodide and orange acridine (PI/OA) staining. The PI red signal revealed cells that had lost membrane integrity (dead cells), over the total number of cells forming the spheroid (all stained in green with OA). The results were represented as estimated cell survival rates.

The migration assay was performed after the exposition of spheroids to MAL-PDT. Spheroids were transferred to a 12-well adherent plate with culture medium, allowing the attachment and migration of the cells that survived to MAL-PDT. Phase contrast microscopy images were taken at different time points after transferring the spheroids.

### 2.5. Flow cytometry

Lyophilized recombinant human TGFβ1 (Peprotech, London, UK) was reconstituted in 10 mM citric acid (Panreac, Barcelona, Spain) at a concentration of 1 μg/ml, with the pH adjusted to 7.4. From this stock solution, different concentrations of TGFβ1 for treatments were prepared in DMEM supplemented with 1% antibiotics (penicillin, 100 U/ml; streptomycin, 100 µg/ml) and no FBS. Cells treated with TGFβ1 (0, 0.1, 1 or 10 ng/ml) for 48 h were then incubated with 0.5 mM MAL in DMEM without FBS for 5 h and immediately analyzed by flow cytometry to quantify the levels of PpIX. Untreated cells (no MAL, no TGFβ1) were used as the unstained control. After the treatments, the cells were trypsinized and centrifuged at 480 g for 5 minutes, the pellets were resuspended in 0.2 ml of PBS and immediately evaluated. The measurement of PpIX emission was obtained using a FC500 flow cytometer (CytoFLEX, 3 lasers, Beckman Coulter, CA, USA) (λ excitation = 405 nm and λ emission = 633 nm). Data were analyzed using the FlowJo X 10.0.7.r2 software.

### 2.6. Indirect immunofluorescence

For indirect immunofluorescence (IF), cells were seeded and grown on glass coverslips until reaching around 70% confluence, subjected to the corresponding treatments and fixed with 3.7% formaldehyde (Sigma-Aldrich, St. Louis, MO, USA) in PBS at 4 ᵒC (Thermo Fisher Scientific Inc., Rockford, IL, USA). Next, cells were washed with PBS and permeabilized with 0.5% Triton X-100 in PBS at room temperature. Coverslips were subsequently incubated with primary antibodies against SMAD2/3 (sc-133098, Lot. L0820, Santa Cruz Biotechnologies, DA, USA) or endoglin (kindly provided by Dr. Botella and obtained from the P4A4 hybridome 20) diluted in 0.5% BSA (Sigma-Aldrich, St. Louis, MO, USA) in PBS for 1 h at 37 ᵒC. After washing with PBS, AF488 goat anti-mouse IgG (Thermo Fisher Scientific Inc.) secondary antibody was added and incubated for 45 minutes at 37 ᵒC. Finally, coverslips were washed again and incubated with Hoechst 33258 (Sigma-Aldrich) for 5 minutes at room temperature for nuclear counterstaining and mounted with ProLong® (Life Technologies, Carlsbad, CA, USA). Slides were observed under the epifluorescence microscope.

### 2.7. Western blot

Protein extracts were prepared using RIPA buffer containing 1% Triton X-100 (bioWORLD, Dublin, OH, USA), phosphatase inhibitor (PhosSTOP EASYpack, Roche, Mannheim, Germany), and protease inhibitors (complete ULTRA tablets Mini EDTA-free EASYpack, Roche, Mannheim, Germany). Protein concentration was determined using the BCA Protein Assay Kit (Thermo Scientific Pierce, Rockford, IL, USA). Protein extracts were diluted in Laemmli buffer mixed with β-mercaptoethanol (Bio-Rad, Hercules, CA, USA) (except for endoglin detection, which was carried out in non-reducing conditions). All protein extracts were heated to 98 ᵒC for 10 minutes. Electrophoresis was performed using acrylamide/bis-acrylamide gels in denaturing conditions (SDS-PAGE) and transferred to polyvinylidene difluoride (PVDF) membranes (Bio-Rad, Hercules, CA, USA), using a Transblot Turbo system (Bio-Rad, Hercules, CA, USA). Membranes were blocked in 5% skimmed milk in 0.1% Tween-20 in TBS (TBS-T). Membranes with proteins were incubated with primary antibodies against SMAD2/3 (sc-133098, Lot. L0820, Santa Cruz Biotechnology, DA, USA), P-SMAD2/3 (ab254407, Lot. GR3427088-3, Abcam, Cambridge, UK), P21 (556431, Lot. 2353387, BD Biosciences, CA, USA), endoglin (kindly provided by Dr. Botella from P4A4 hybridome), GAPDH (ab181602, Lot. GR217575-9, Abcam, Cambridge, UK), and vinculin (V9131, Lot. 0000233674, Sigma-Aldrich, St. Louis, MO, USA). After washing with TBS-T, membranes were incubated with the corresponding peroxidase-conjugated secondary antibodies (HRP-Goat anti-rabbit IgG and HRP-Goat anti-mouse IgG, Thermo Fisher, Rockford, IL, USA). Protein bands were visualized by chemiluminescence (ECL Plus Kit, Amersham, Little Chalfont, UK) using the high-resolution ChemiDocTR XRS+ system (Bio-Rad) and digitized using Image Lab version 6.1 software (6.1 version, Bio-Rad Software, Hercules, CA, USA). In all cases, protein expression levels were normalized to a loading control.

### 2.8. 5-ethynyl-2-desoxyuridine (EdU) incorporation assay

cSCC cells were grown on coverslips and treated with the different CM described above for 48 h. Cells were then incubated for 3 h with EdU, fixed with formaldehyde and further processed following the instructions of the click-iT EdU kit Alexa Fluor 555 (Thermo Fisher Scientific Inc., Rockford, IL, USA). Samples were mounted with Prolong Gold antifade reagent and evaluated under the epifluorescence microscope.

### 2.9. Measurement of secreted TGFβ1 and soluble endoglin

When cells cultured in 24-well plates reached 80% confluence, media were replaced with 250 µL of phenol red-free DMEM (Thermo Fisher Scientific Inc., HyClone, Rockford, IL, USA) supplemented with 1% FBS and 1% antibiotics, containing NAC or raloxifene if appropriate. After 24 h, the medium was harvested, centrifuged at 480 g for 5 min and the supernatant was stored at -80 ᵒC until analyzed. Cells that remained attached to the surface of the plate were lifted and counted using a TC20TM automated cell counter (BioRad) to normalize the TGFβ1 measurements. For TGFβ1 quantification in culture media or in serum samples isolated from mouse blood, latent TGFβ1 was activated for detection by incubating the samples with 1 M hydrochloric acid, followed by neutralization by adding a solution containing 1.2 M sodium hydroxide and 0.5 M HEPES, and the concentration of TGFβ1 was measured using the Quantikine® ELISA Human TGFβ1 Immunoassay (R&D Systems, Minneapolis, MN, USA) kit. To detect and quantify soluble endoglin in fibroblast-derived CM, the Quantikine® ELISA Human endoglin Immunoassay (R&D Systems, Minneapolis, MN, USA) kit was used.

### 2.10. Endoglin silencing with siRNA and Treatment with recombinant endoglin

ENGsiRNA (s4677) and the negative control (AM4611) (both from Ambion, Life Technologies, CA, USA) were transiently introduced into T205A CAF cultures at approximately 80% confluence using Lipofectamine™ LTX Reagent with PLUS™ Reagent (Invitrogen, CA, USA) following the standard protocol provided by the manufacturer. Endoglin silencing was validated by Western blot using protein extracts obtained 96 h after silencing.

Lyophilized recombinant human endoglin (R&D Systems, Minneapolis, MN, USA) was reconstituted in PBS containing 0.1% BSA to prepare a 250 µg/ml stock solution. Subsequent dilutions were prepared in DMEM containing 1% FBS and 1% antibiotics and added to the cultures 24 h prior to media collection for TGFβ1 quantification.

### 2.11. Photocarcinogenesis induction in SKH-1 mice by chronic exposure to ultraviolet light

Female SKH-1 hairless mice (5-7 weeks old) were purchased from Charles River Laboratories (Barcelona, Spain) and maintained at the animal facility of the National Center of Biotechnology (CNB, Madrid, Spain), in a room at 24 ± 2 ᵒC, with 12 h light/12 h dark cycles, 50 ± 10% relative humidity, and food and water *ad libitum*. All animal procedures were approved by the Ethics Committee of Consejo Superior de Investigaciones Científicas (CSIC, Spain) and Comunidad Autónoma de Madrid (CAM, Consejería de Medio Ambiente; PROEX 165.0/20) and carried out in compliance with the guidelines in RD53/2013 (Spain).

A total of 45 mice were divided into four groups: UV (n = 15), UV + PDT (n = 10), UV + NAC (n = 10), and UV + NAC + PDT (n = 10). All mice were subjected to three sessions per week of ultraviolet light (UV) irradiation to induce the development of skin tumors as previously described 21. Irradiation was stopped when approximately 50% of the animals presented at least one visible lesion above 8 mm^3^ (time point designated as day 0, corresponding to 117 days after the onset of UV irradiation, with an accumulated UV dose of 10.53 J·cm^-2^). The UV source used, which has been developed by our laboratory, is equipped with 6 UV fluorescent tubes (Phillips TL UV, 20 W). The spectrum of this source was registered with a Solatell Sola Scope-I radiometer (Croydon, UK). It ranges from 270 to 380 nm, with a maximum peak at 312 nm and approximately 52% UVB, 47% UVA and 1% UVC radiation. The light source was set at 15 cm distance from the dorsal surface of the mice.

Tumor size was measured with an automatic caliper once a week since day 0 and tumor volume was estimated as follows: Tumor width*Tumor length*(the lowest measurement/2). This formula was employed due to the difficulty of obtaining a reliable measurement of the depth of cutaneous tumors 22.

For hematological analysis, blood was collected from the submandibular vein into microtainer tubes containing a separating gel (BD Biosciences, CA, USA). Blood extraction was carried out at three different time points: day -117 (before UV irradiation starts), day 0 (before treatment application) and day 42 (before end point). After blood extraction, samples were centrifuged at 6000 g for 15 minutes at 4ᵒC and serum was stored at -80ºc until analyzed.

### 2.12. Mouse treatments

NAC treatment was administered to the animals belonging to the groups UV+NAC and UV+NAC+PDT. NAC cream formulation was kindly provided by Dr. Abarca-Lachen (Lachen Laboratory, Huesca, Spain) and consisted of NAC (10%), Transcutol P (20%) and emulsifier Neo PCL O/W csp (50 g). A defined amount of NAC cream (15 mg) was topically applied and homogeneously distributed on the back skin of each mouse 5 times a week, starting at the end of UV irradiation (day 0) and until the end of the experiment (day 42).

For PDT administration, 25 mg of MAL-containing cream (Metvix®, Galderma, Zug, Switzerland) were topically applied on the back skin. After 3 h incubation in the dark, Metvix® excess was eliminated with absorbent cotton soaked in 0.9% NaCl. Subsequently, mice were anesthetized with a mixture of 1.5-2% isofluorane in oxygen at 3 l/min using an isofluorane chamber (Ecuphar, Barcelona, Spain) and kept at room temperature throughout the exposition to red light (636 nm). For the irradiation, the lamp (Aktilite®, 50 Mw·cm^-2^) was located at 5 cm distance from the dorsal surface of the mice. The dorsal surface of each mouse was irradiated with 6 J·cm^-2^ in the first PDT administration (day 14) and 9 J·cm^-2^ in the second PDT administration (day 28). On day 42 after terminating the UV irradiation, mice were euthanized with CO_2_ and the dorsal skin was surgically removed and collected for further analysis.

### 2.13. Hematoxylin-eosin staining and immunohistochemistry

Skin samples were fixed in 10% formalin and embedded in paraffin. Vertical sections (5 µm thickness) were obtained, mounted on glass slides and stained with hematoxylin-eosin following the usual procedure. For immunohistochemistry (IHC), antigen retrieval was achieved by boiling the sections in citrate buffer (0.25% citric acid and 0.038% sodium citrate in water, pH=6) using a microwave oven. After cooling the samples to room temperature, endogenous peroxidase activity was inhibited with 3% hydrogen peroxide (Panreac, Barcelona, Spain) in methanol. Non-specific binding sites were blocked with non-immune serum (Dako, Agilent Technologies, Santa Clara, CA, USA) for 30 minutes at room temperature, followed by overnight incubation at 4 °C with primary antibodies against P53 (Cell signaling, Technology, Inc, Danvers, MA, USA) or PCNA (Calbiochem, St. Louis, MO, USA) diluted in 2% BSA in PBS. Next, the sections were incubated with the secondary antibodies conjugated with streptavidin-peroxidase (Cell Signaling Tecnology, Inc, Danvers, MA, USA) for 30 min at room temperature. Color was developed using 3-amino-9-ethylcarbazole solution as chromogen (DAB, Vector laboratories, Burlingame, CA, USA) and Harris hematoxylin counterstaining was performed following the manufacturer's protocol. Finally, the sections were dehydrated in a series of increasing concentrations of ethanol and mounted with DePeX (Serva, Heidelberg, Germany).

### 2.14. Microscopy and image analysis

Fluorescent imaging was performed using an Olympus BX61 fluorescence microscope (Olympus Corporation, Shinjuku, Tokyo, Japan) equipped with a pE-300LITE LED lamp and filter sets for various fluorescence microscopy applications. OA and AF488 were detected under blue light irradiation (450-490 nm, BP 490 filter); green light irradiation (570-590 nm, DM 590 filter) was used for PI and EdU detection; and UV irradiation (360-370 nm, UG-1 filter) for Hoechst-33258. All quantifications (mean fluorescence intensity) were performed on raw images taken under equivalent exposure conditions and never on adjusted ones. In addition, for mean fluorescence intensity quantification, equal numbers of cells were seeded to standardize the level of confluence and avoid confluence-dependent variations at the time point of evaluation. In the spheroid migration assays, the diameter of the area covered by cells was measured using the FIJI software (ImageJ, version 1.53 NIH, USA). The percentage of positive cells resulting from EdU incorporation or IHC assays was estimated in 400x magnified images using the FIJI cell counter tool.

### 2.15. Data representation and statistical analysis

All experiments included at least three technical and three biological replicates. Statistical analyses were performed with GraphPad Prism software (version 8.01, GraphPad Software Inc., San Diego, CA, USA). Outliers were identified and excluded using the ROUT method (Q value = 1%). Bar plots show mean ± standard error of the mean (SEM). The normality of the data was tested using the Shapiro-Wilk normality test. Statistical differences among three or more groups were analyzed using the one-way ANOVA test with the Tukey post-hoc test. Statistical significance was set at p<0.05, p<0.01 and p<0.001, as indicated in figure legends.

## 3. Results

### 3.1. The induction of PDT resistance in A431 cells exposed to CAF-derived CM is abolished as a result of the treatment of CAFs with NAC or raloxifene

**Figure 1:**
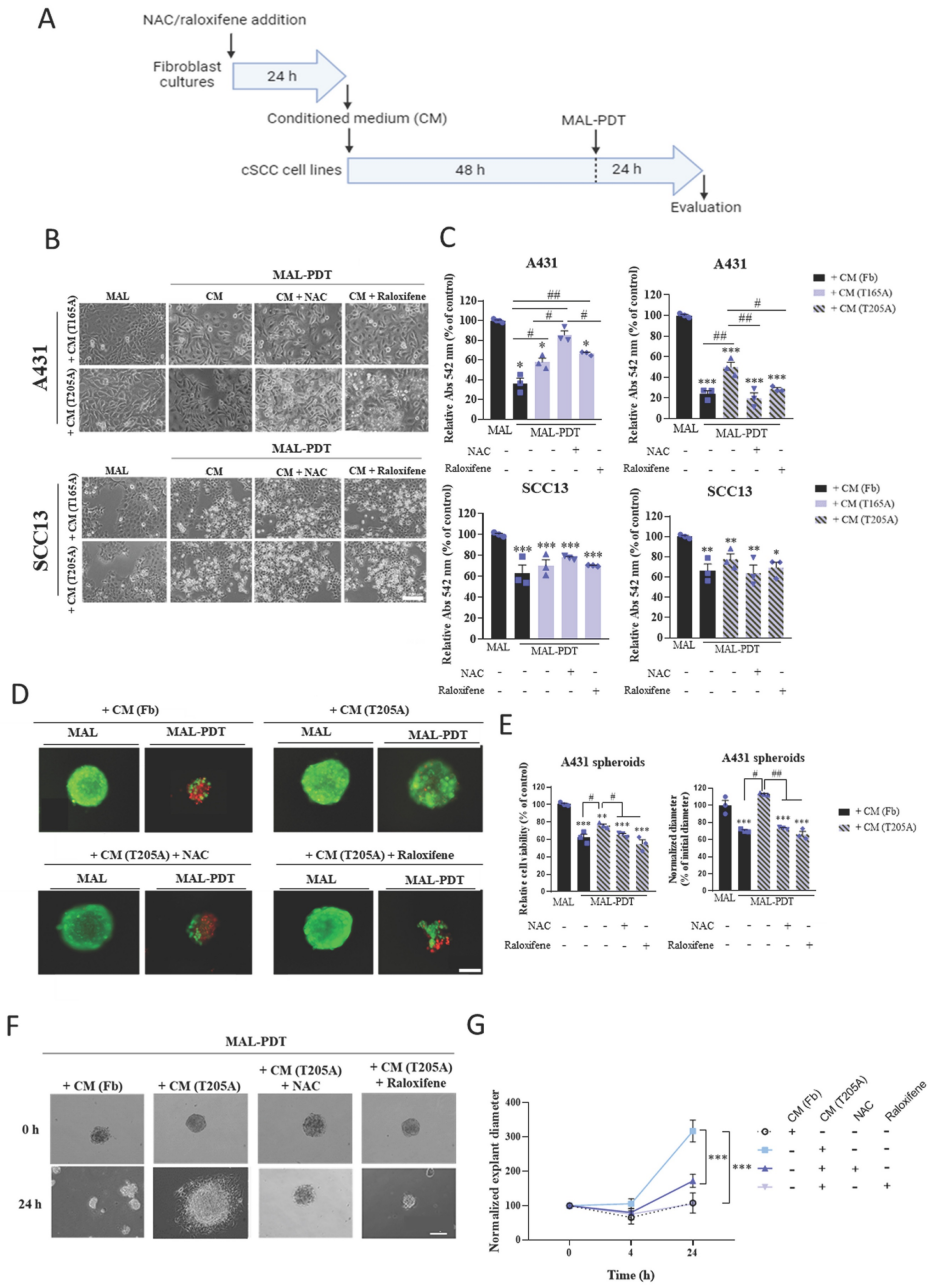
Effect of CAF treatment with NAC and raloxifene in the response to PDT of cSCC cultures exposed to CAF-derived CM. (A) Schematic timeline of the *in vitro* experimental protocol. (B) Phase contrast images illustrating the morphological changes in the morphology directly induced by the incubation of CAF-derived CM. Scale bar: 50 µm. (C) Cell viability rates of A431 and SCC13 cells treated with MAL-PDT (0.5 mM of MAL and red light dose of 9.1 J·cm^-2^) in the presence of each type of CM. The results of the MTT assay are relativized to Fb-derived CM condition with no irradiation. (D) Representative fluorescence microscopy images of A431 spheroids. Alive cells are visualized in green under blue light irradiation (450-490 nm); and dead cells in red under green light irradiation (570-590 nm). Scale bar: 200 µm. (E) Cell viability and size of A431 spheroids in response to MAL-PDT (0.5 mM of MAL, red light dose of 12.2 J·cm^-2^) in the presence of CM from Fb and T205A, pre-treated or not with NAC or raloxifene. Cell viability was evaluated by PI/OA assay. Size was estimated by measuring the diameter of the spheroid in the images taken 24 h after MAL-PDT. (F) Representative phase contrast microscopy images of A431 explants at 0 h and 24 h time points. Scale bar: 200 µm. (G) Measurement of the diameter of A431 explants in response to MAL-PDT in the presence of CM from Fb and T205A pre-treated or not with NAC or raloxifene. A time course (0 h, 4 h and 24 h) of the evolution of the explant diameter is shown. Error bars denote ± S.E.M. (n = 3, one-way ANOVA: statistical comparison to MAL-PDT + CM (T205A) condition at 24 h: *<0.05, **p<0.01 and ***p<0.001).

TGFβ1 secreted by CAFs present in the tumor microenvironment induces resistance to different therapies, in particular to PDT in cSCC cells as it was previously demonstrated by our group 23. We hypothesized that NAC and raloxifene could contribute to reduce TGFβ1 levels in the tumor microenvironment and therefore, combat short term resistance to PDT. To test this hypothesis, two different CAF cultures (T165A and T205A) were treated with 0.1 mM NAC or 0.2 mM raloxifene for 24 h before collecting the CM that will be subsequently added to cSCC cells prior to PDT. Fb were grown in parallel to collect Fb-derived CM for the sake of comparison. Next, two cSCC cell lines (A431 and SCC13) were exposed to the different types of CM for 48 h and subsequently subjected to MAL-PDT. The response to PDT was assessed in terms of cell survival using the MTT assay. A431 cells treated with T165A-derived CM displayed resistance to PDT and the resistance was not reverted by the exposition to T165A-derived CM generated in the presence of NAC or raloxifene. Importantly, T205A-derived CM also induced resistance of A431 cells to MAL-PDT but this response was prevented when CM was collected from T205A CAFs upon treatment with NAC or raloxifene (Figure 1A, 1B, 1C). The concentrations of NAC and raloxifene used to carry out these experiments were selected as non-harmful conditions according to dose-response assays Figure S1A).

To rule out that the antioxidant properties of NAC and raloxifene remaining in the CM were directly interfering with the toxicity of PDT that is mediated by the induction of reactive oxygen species (ROS) production, we compared the response to MAL-PDT of A431 cells exposed or not to NAC or raloxifene and observed similar levels of lethality (Figure S1B). In addition, the exposition of A431 cells to Fb-derived CM collected in the presence or absence of NAC or raloxifene did not interfere with the toxicity of PDT (Figure S1B). These data demonstrate that NAC and raloxifene do not reduce PDT effectiveness by directly scavenging ROS and also confirm that Fb-derived CM does not induce resistance to PDT in A431 cells. Notably, SCC13 cells did not develop resistance to PDT in response to CAF-derived CM, and no differential effect was detected upon pretreatment of CAFs with NAC or raloxifene (Figure 1A, 1B, 1C, S2A). The corresponding control treatments were carried out in all cases: untreated cells as well as cells treated only with MAL (0.5 mM, 5 h), or only with red light (9.1 J·cm^2^). No cell toxicity was detected in any of the control conditions (data not shown). In addition, as a proof of concept, we have collected evidence supporting the direct effect of TGFβ1 inducing a decrease in the production of the endogenous photosensitizer PpIX in cSCC cell lines incubated with MAL (Figure S2B). The results revealed that the production of PpIX upon incubation with MAL is reduced in A431 cells in response to the treatment with TGFβ1 in a dose-dependent manner. Interestingly, in SCC13 cells, the lowest concentration of TGFβ1 used (0.1 ng/ml) did not induce any reduction of the PpIX levels compared to cells treated only with MAL. As the effect of TGFβ1 was much more potent in A431 cells compared to SCC13 cells (Figure S2B), the data suggest that this could be linked with the reduced toxicity of PDT in A431 cells exposed to CAF-derived CM.

We then aimed at addressing if the capacity of NAC and raloxifene to abolish resistance to PDT induced by CAF-derived CM in bidimensional cultures also affected 3D *in vitro* models, which more faithfully reproduce the physiological conditions operating in tumors *in vivo*. Spheroids were formed using A431 cells and treated following a similar protocol to that used for bidimensional cultures. Briefly, A431 spheroids were incubated with CM obtained from T205A CAFs in the presence or absence of NAC or raloxifene and subsequently treated with MAL-PDT. PI/OA staining was performed to estimate the cell viability rate using fluorescence microscopy. The results observed recapitulated the data obtained in the monolayer cultures. The toxicity of PDT was significantly reduced in the presence of T205A-derived CM alone, revealing a resistance response similar to that observed in bidimensional cultures. Moreover, CAF pre‑treatment with NAC or raloxifene abolished this resistance effect (Figure 1D, 1E).

The size of the spheroids was estimated by measuring the diameter 24 h after MAL-PDT in each experimental condition, revealing that the spheroids subjected to PDT upon exposure to T205A‑derived CM were larger than those treated with PDT upon exposure to T205-derived CM obtained in the presence of NAC or raloxifene (Figure 1D, 1E). Furthermore, a migration assay was carried out 24 h after PDT to investigate the ability of the cells to migrate forming explants. To this end, the spheroids were transferred to 12-well plates upon treatment with Fb-derived CM or T205A-derived CM obtained in the presence or absence of NAC or raloxifene, followed by PDT administration. A follow-up of the explant evolution was carried out by sequentially taking phase contrast images at different time points (0, 4 and 24 h after the transference to the plate). At 4 h, most of the living spheroids slightly decreased in size and started to adhere to the well. After 24 h from MAL-PDT, spheroids containing living cells appeared completely adhered to the plate, forming explants, while spheroids made up by dead cells remained in suspension. The size of the explants after PDT indicated increased migratory capacity induced by the pre‑treatment with T205A-derived CM, compared to Fb-derived CM. In addition, the pre‑treatment with T205A-derived CM obtained in the presence of NAC or raloxifene in similar migratory values after PDT than those of the Fb-derived CM. The same results were obtained for both doses of PDT that were tested (Figure 1F, 1G, S2C). These results support that CAF-derived CM is able to induce resistance to PDT in A431 cells increasing both cell survival and the migratory capacity, and that NAC and raloxifene treatments are able to abolish the induction of resistance to PDT in 3D culture models.

### 3.2. P-SMAD translocation into the nucleus is associated with cell cycle arrest of A431 cells in response to CAF-derived CM

**Figure 2:**
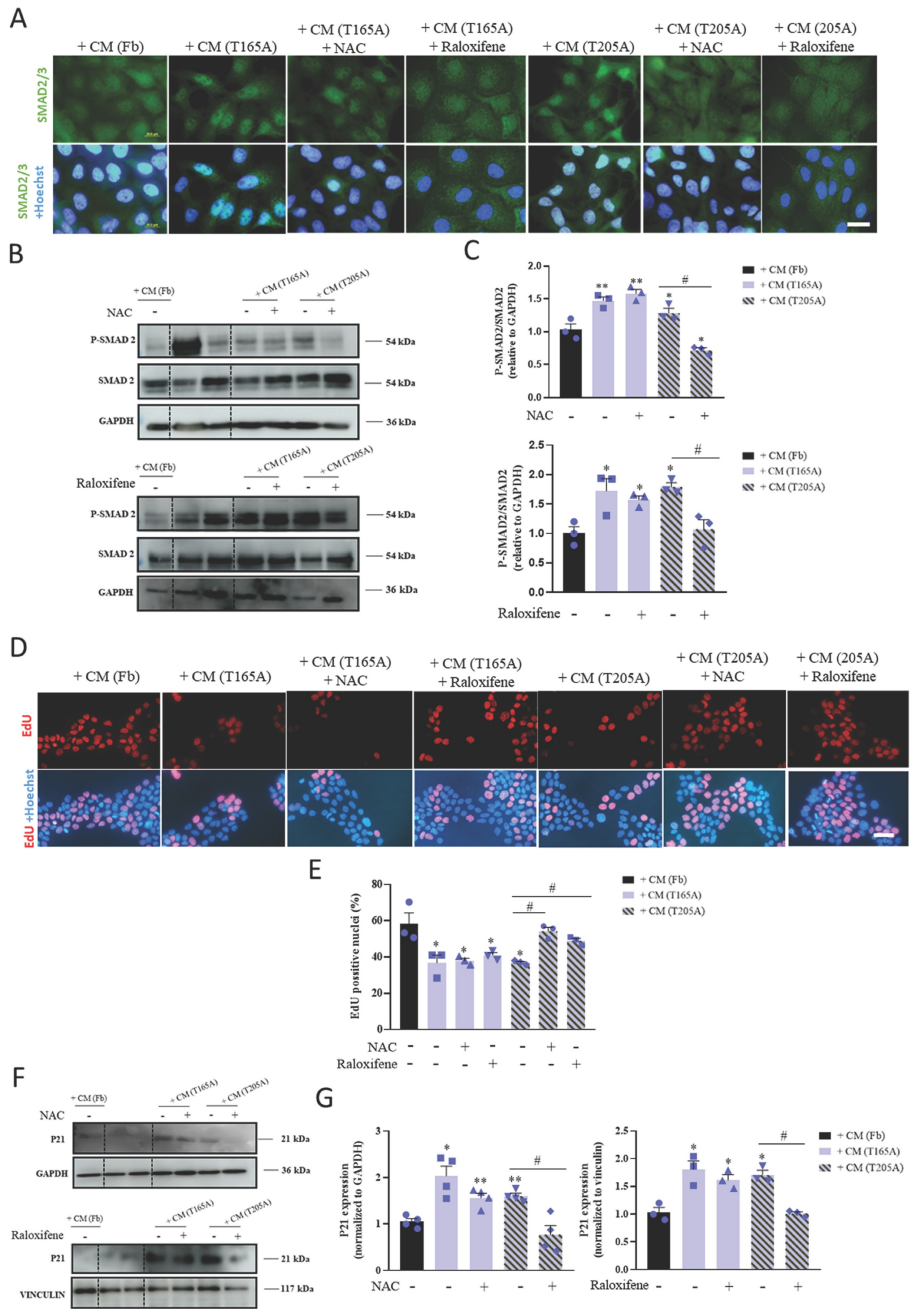
Effect of CM from CAFs in the presence or absence of NAC or raloxifene on the TGFβ1/SMAD2/3 pathway in A431 cells. (A) Localization of SMAD2/3 analyzed by IF. Hoechst-33258 (Ho) was used for nuclear counterstaining. Scale bar: 20 µm. (B) P-SMAD 2 and SMAD2 expression analysis by Western blot in A431 cells. Bands between the dotted lines (lanes 2 and 3) correspond to protein extracts of no interest for the present work. Western blot images are representative of n = 3 independent experiments. (C) Analysis of the expression of P-SMAD2/SMAD2 ratio in A431 cells using ImageLab software. All the results are relativized to Fb-derived CM treatment. Error bars denote ± S.E.M. (n = 3, one-way ANOVA, statistical comparisons to Fb condition: *<0.05, **p<0.01 and ***p<0.001; multiple comparisons: #p<0.05, ##p<0.01 and ###p<0.001). (D) EdU labelling detection in A431 cells using fluorescence microscopy. Upper row: fluorescence microscopy images revealing EdU incorporation. Lower row: nuclear counterstaining with Hoechst-33258. All images were taken with the same magnification. Scale bar: 50 µm. (E) Quantification of the percentage of EdU positive nuclei in A431 cells. All the results are relativized to Fb-derived CM treatment. Error bars denote ± S.E.M. (n = 3, one-way ANOVA, statistical comparisons to Fb condition: *<0.05, **p<0.01 and ***p<0.001; multiple comparisons: #p<0.05, ##p<0.01 and ###p<0.001). (F) Expression of P21 analyzed by Western blot in A431 cells. Bands between the dotted lines (lanes 2 and 3) correspond to protein extracts of no interest for the present work. Western blot images are representative of n = 3 independent experiments. (G) Analysis of the expression of P21 in A431 cells using ImageLab software. All the results are relativized to Fb-derived CM treatment. Error bars denote ± S.E.M. (n = 3, one-way ANOVA, statistical comparisons to Fb condition: *<0.05, **p<0.01 and ***p<0.001; multiple comparisons: #p<0.05, ##p<0.01 and ###p<0.001).

In order to understand the mechanisms by which CAF-derived CM was differentially inducing resistance in A431 compared to SCC13 cells, we hypothesized that TGFβ1 secreted by CAFs may be driving this effect by exerting cytostatic activity. We first investigated the effect of CAF-derived CM on the TGFβ1/SMAD2/3 pathway in A431 and SCC13 cells. To this end, we treated the cells with CM for 48 h and studied the pattern of expression of SMAD2/3 by immunofluorescence as well as the levels of the total and phosphorylated forms of SMAD2 by Western blot. Nuclear localization of SMAD2/3 was observed in A431 cells in response to CAF-derived CM (both from T165A and T205A CAFs), which is consistent with P‑SMAD2/3 translocation into the nucleus. A similar pattern resulted after treating A431 cells with T165A-derived CM + NAC or T165A-derived CM + raloxifene. On the contrary, the nuclear signal was not evident after treating A431 cells with Fb-derived CM, T205A-derived CM + NAC or T205A-derived CM + raloxifene (Figure 2A). No nuclear localization of SMAD2/3 was detected in SCC13 cells exposed to CAF-derived CM (Figure S3A). Semiquantitative analyses revealed an increase in P-SMAD2 expression levels in A431 cells treated with CAF‑derived CM compared to Fb-derived CM. However, in the presence of NAC or raloxifene, this increase was completely abolished. On the other hand, no significant differences were detected in the level of expression of SMAD2 and P-SMAD2 in SCC13 cells (Figure 2B, 2C, S3B, S3C).

One of the widely reported effects of the nuclear translocation of P-SMAD2/3 is the capability to induce cell cycle arrest, preventing cell proliferation (12. Based on this, we next evaluated the effect of CAF-derived‑ CM collected in the presence or absence of NAC or raloxifene on cell cycle dynamics of A431 cells using the EdU incorporation assay. As shown in Figure 2D and 2E, the incorporation of EdU in A431 cells exposed to CAF-derived CM was significantly lower than in cells treated with Fb-derived CM. The treatment of CAFs with NAC or raloxifene significantly rescued the values of the incorporation of the thymidine analogue in A431 cells. Nonetheless, SCC13 cells did not show changes in the number of EdU positive nuclei in any of the conditions tested Figure S3D, S3E). Thus, these results suggest that the treatment of CAFs with NAC and raloxifene prevent the cell cycle arrest induced by CAF-derived CM in A431 cells.

Previous works using different experimental systems identified a role for the P21 protein, a well-known inhibitor of the cell cycle progression, in mediating the cell cycle arrest induced by TGFβ1 (24. Thus, to dig deeper into the intracellular cascades underlying the cell cycle arrest observed in our model system, we evaluated the expression of P21 by Western blot. The results revealed higher levels of P21 expression in A431 cells treated with CAF-derived CM compared to A431 cells treated with Fb-derived CM. Importantly, the treatment of CAFs with NAC and raloxifene effectively prevented the CAF-derived CM-induced overexpression of P21 in A431 cells (Figure 2F, 2G). In addition, in SCC13 cells, CAF-derived CM did not induce any changes in the levels of P21 Figure S3F, S3G). Overall, these results suggest the involvement of P21 in the induction of resistance to PDT in A431 cells.

### 3.3. The production of TGFβ1 by CAFs can be modulated by NAC and raloxifene which regulate the expression of the TGFβ1 co‑receptor endoglin

**Figure 3:**
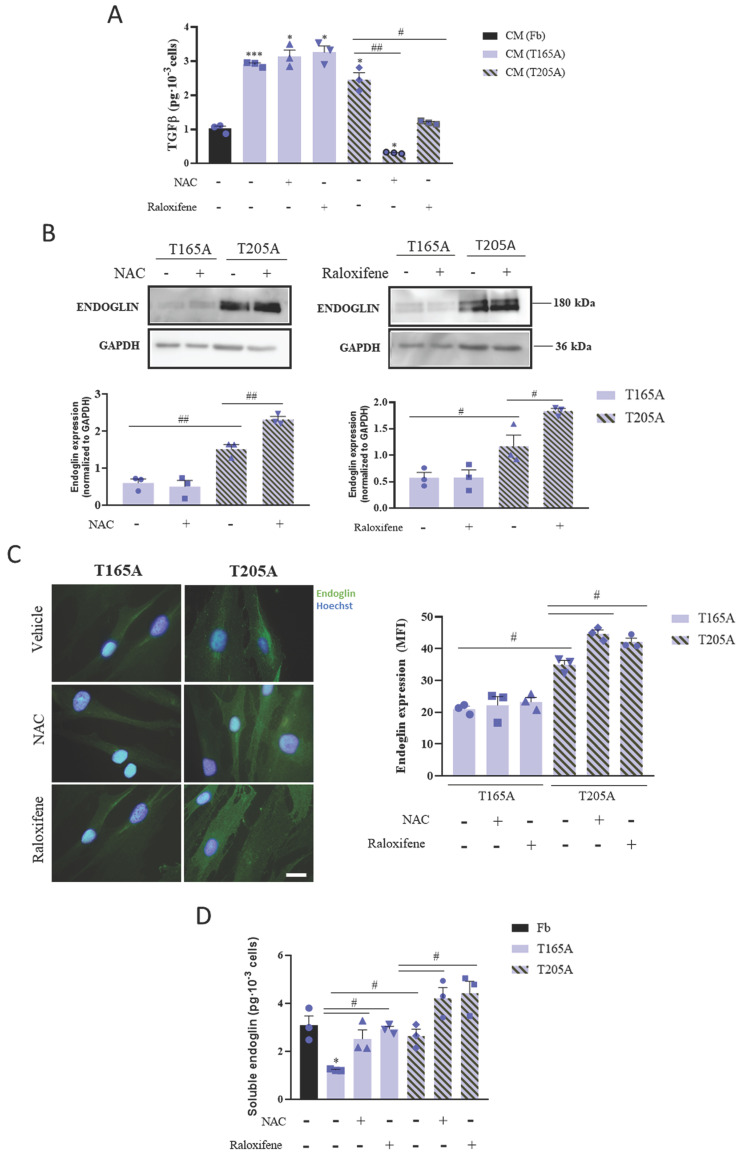
Effect of NAC or raloxifene treatment on TGFβ1 secretion and endoglin expression by CAFs. (A) Secretion of TGFβ1 produced by Fb, T165A and T205A in the presence or absence of NAC or raloxifene. ELISA assay was performed to quantify the levels of TGFβ1 secreted to the culture medium collected 24 h after treatments. (B) Expression of endoglin analyzed by Western blot. Western blot images are representative of n = 3 independent experiments. Error bars denote ± S.E.M. (n=3, one-way ANOVA, multiple comparisons: #p<0.05, ##p<0.01 and ###p<0.001). (C) Left panel: fluorescence microscopy images of endoglin expression. Counterstain with Hoechst-33258 was used as a control for nuclei visualization. All images were taken with the same magnification. Right panel: quantification of endoglin expression is represented as the mean fluorescence intensity (MFI). (D) Analysis of soluble endoglin cleaved from the membrane in Fb, T165A and T205A in the presence or absence of NAC or raloxifene. ELISA assay was performed to quantify the levels of cleaved endoglin in the culture medium collected 24 h after treatments. Error bars denote ± S.E.M. (n = 3, one-way ANOVA, statistical comparisons to Fb condition: *<0.05, **p<0.01 and ***p<0.001; multiple comparisons: #p<0.05, ##p<0.01 and ###p<0.001).

In order to decipher the molecular mechanisms by which the treatment with NAC or raloxifene affects CAFs and ultimately prevents the induction of resistance to PDT in cSCC cells, we first investigated whether the production of TGFβ1 by CAFs changed in response to the compounds. For this, CAFs and Fb were exposed to NAC or raloxifene for 24 h prior to the collection of culture media, and TGFβ1 levels were analyzed by ELISA. As expected, the levels of TGFβ1 secreted by CAFs were higher than those produced by Fb (Figure 3A). Moreover, while NAC and raloxifene decreased the TGFβ1 levels secreted by T205A, no changes were detected in the production of TGFβ1 by T165A in response to NAC or raloxifene (Figure 3A).

Previous studies have addressed how NAC and raloxifene can modulate the expression of the TGFβ1 co‑receptor endoglin. This co-receptor has been implicated in the inhibition of TGFβ1 signaling and also participates in carcinogenesis (19. In light of this knowledge, we next investigated endoglin expression in T165A and T205A cultured CAFs, exposed or not to NAC or raloxifene for 24 h. We detected significantly lower basal levels of endoglin in T165A compared to T205A, and significant upregulation of endoglin in T205A CAFs upon treatment with NAC or raloxifene (Figure 3B, 3C).

Previous studies have described that endoglin also has a soluble form, generated as a consequence of the proteolytic cleavage of the extracellular domain from membrane endoglin. Thus, we analyzed the effect of the treatment with NAC and raloxifene for 24 h on the presence of soluble endoglin in the CAFs culture medium. The ELISA results confirmed that soluble endoglin levels were consistently increased by NAC and raloxifene in both T165A and T205A, compared with the corresponding non-treated controls (Figure 3D). Interestingly, the levels of soluble endoglin in the culture medium from T165A upon exposure to NAC and raloxifene reached similar values to those of untreated T205A CAFs (Figure 3D).

As no effect of NAC or raloxifene on the secretion of TGFβ1 by T165A CAFs was detected, we selected T205A CAFs for further experimentation to investigate the participation of endoglin to limit the production of TGFβ1 in response to NAC.

### 3.4. Endoglin levels in CAFs determine the sensitivity of cSCC cells to PDT

**Figure 4:**
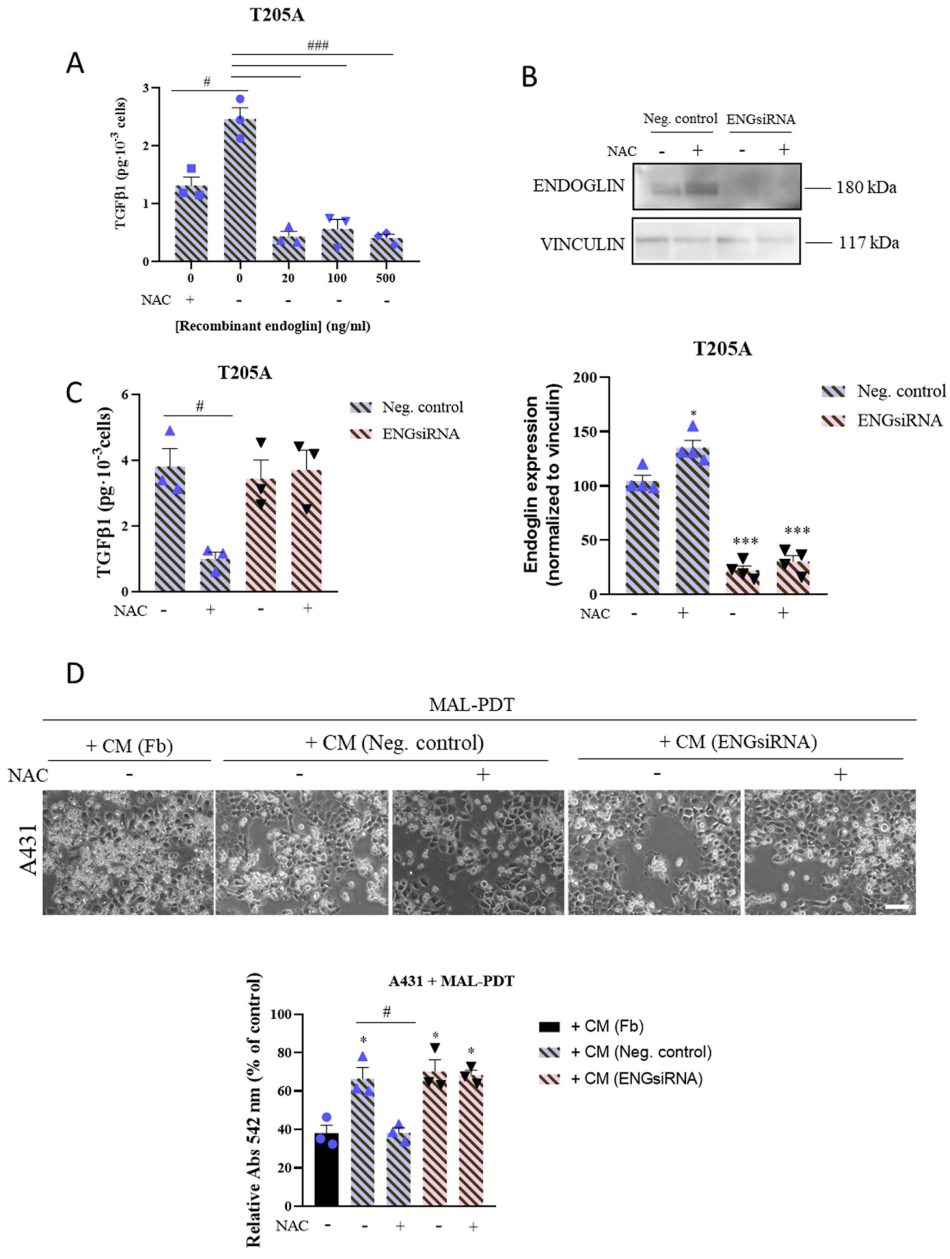
Effect of CAF endoglin expression levels on TGFβ1 secretion and on the induction of resistance to PDT in A431 cells. (A) Secretion of TGFβ1 produced by T205A after treatment with increasing concentrations of recombinant endoglin. ELISA assay was performed to quantify the levels of secreted TGFβ1 to the culture medium collected 24 h after treatments. (B) Expression of endoglin analyzed by Western blot in T205A CAFs after transfection with the negative control (NC) and a siRNA targeting endoglin. Western blot images are representative of n = 3 independent experiments. Error bars denote ± S.E.M. (n = 4, one-way ANOVA: *p<0.05, **p<0.01 and ***p<0.001). (C) Secretion of TGFβ1 produced by T205A CAFs after transfection with NC or ENGsiRNA. ELISA assay was performed to quantify the levels of secreted TGFβ1 to the culture medium collected 24 h after treatment with NAC (D) Effect of the pre-treatment with NAC of transfected T205A in the CAF-derived CM on the response of bidimensional A431 cell cultures to PDT. Upper row: phase contrast images illustrating the morphological changes in A431 cells after treatments. Scale bar: 50 µm. Lower row: cell viability rates of A431 cells treated with MAL-PDT (0.5 mM of MAL and red light dose of 9.1 J·cm^-2^) in the presence of T205A CM after transfection. Error bars denote ± S.E.M. (n = 3, one-way ANOVA, statistical comparisons to Fb condition: *<0.05, **p<0.01 and ***p<0.001; multiple comparisons: #p<0.05, ##p<0.01 and ###p<0.001).

To validate the involvement of endoglin in the mechanism of action of NAC to prevent PDT resistance in cSCC cells, we first analyzed the effect of the addition of recombinant endoglin on the secretion of TGFβ1 by T205A CAF cultures. We treated T205A CAFs with increasing concentrations of recombinant endoglin for 24 h. T205A CAFs were treated with 0.1 mM NAC as a positive control for the decrease of TGFβ1 levels. The results revealed that the levels of secreted TGFβ1 were inversely correlated with the concentration of endoglin in the culture medium (Figure 4A).

On the other hand, we silenced the expression of endoglin in T205A CAFs using siRNA technology in order to confirm the role of this protein in preventing the induction of resistance to PDT in A431 cells. We first validated by Western blot the reduction in the expression of endoglin upon silencing, in the presence or absence of NAC. Indeed, we obtained a 70% reduction in the levels of endoglin in T205A‑ENGsiRNA compared to the non-silenced control, and endoglin levels remained unchanged in response to 0.1 mM NAC (Figure 4B). Then, we evaluated the levels of TGFβ1 secreted by T205A‑ENGsiRNA compared to non-silenced control cells. The ELISA results revealed that T205A‑ENGsiRNA were irresponsive to NAC, as the significant decrease of TGFβ1 levels in response to NAC treatment was only observed in the T205A CAFs non-silenced control (Figure 4C).

Finally, we used CM from T205A‑ENGsiRNA or the corresponding non‑silenced control, exposed or not to NAC, to treat A431 cells for 48 h prior to the administration of MAL-PDT. The results indicated that CAF-derived CM efficiently induced resistance to PDT, independently of endoglin silencing. Noteworthy, the effect of NAC to abolish the induction of resistance to PDT was only observed when using CM from non-silenced T205A CAFs, supporting a relevant role of endoglin to overcome PDT resistance in A431 cells (Figure 4D).

### 3.5. Topical treatment with NAC enhances the efficacy of PDT in a mouse model of skin cancer

**Figure 5:**
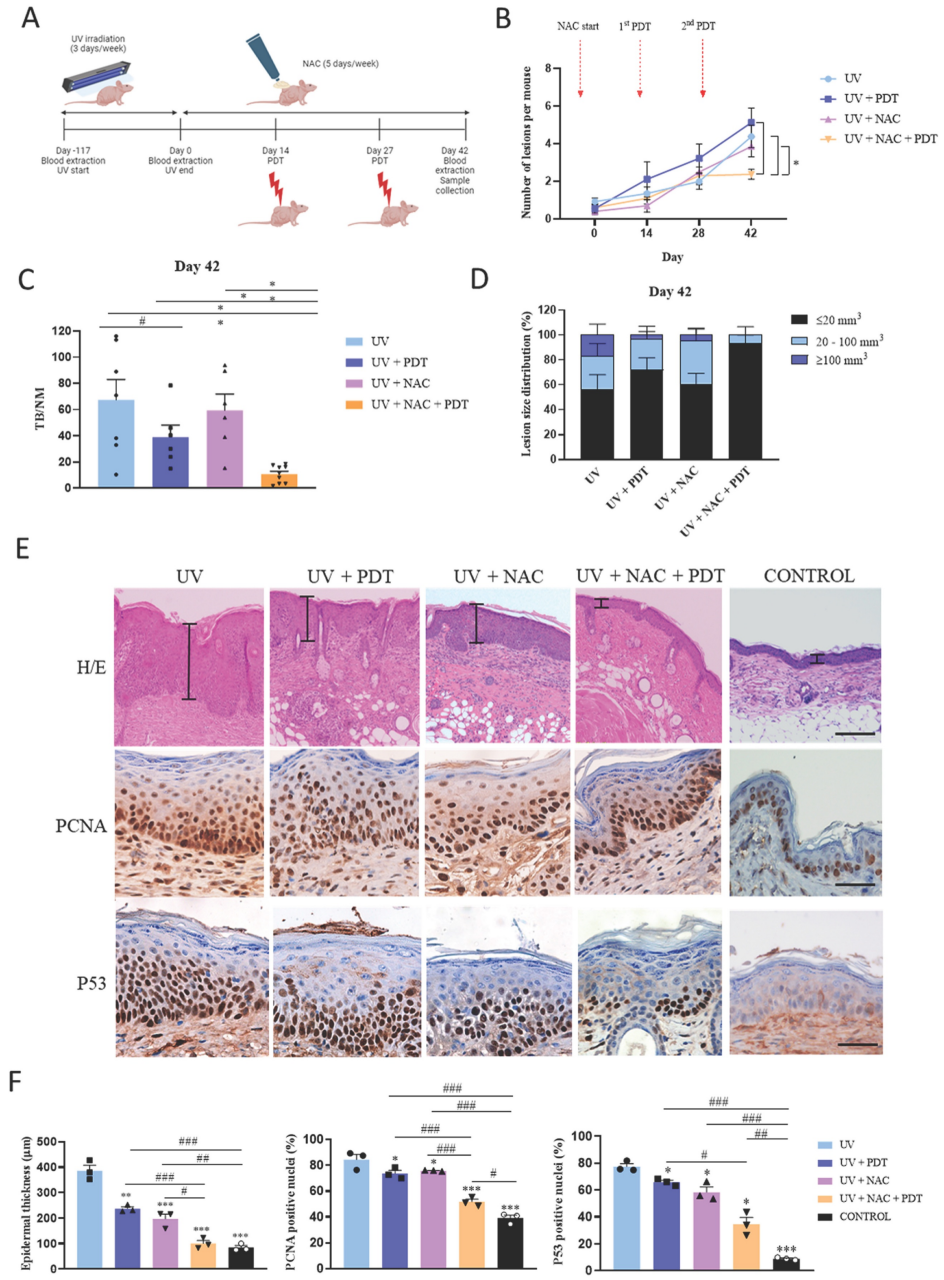
Combined treatment with NAC + PDT applied to SKH-1 mice chronically exposed to UV and histopathological analyses. (A) Schematic timeline of the animal experimental protocol. Day 0 indicates the end of UV irradiation (after 117 days, reaching an accumulated dose of 10.53 J·cm^-2^) and the onset of NAC treatment. First PDT dose (day 14): Metvix®+ 6 J·cm^-2^ red light; second PDT dose (day 27): Metvix®+ 9 J/cm^2^ red light. (B) Representation of the total number of lesions per mouse at each time point. (C) Normalized tumor burden (TB/NM) at day 42 (end point). (D) Distribution of existing tumors categorized according to estimated volume within the total number of lesions in each group at day 42. Error bars denote ± S.E.M. (n = between 9 and 13 per group). (E) Representative images of mouse dorsal skin samples (hematoxylin-eosin staining, scale bar: 200 µm); and PCNA and P53 positivity in mouse dorsal skin samples (immunohistochemistry, scale bar: 50 µm). (F) Epidermal thickness analysis of mouse dorsal skin samples; and analysis of PCNA and P53 expression (n = 3 per group, one-way ANOVA: statistical comparisons to UV condition; *<0.05, **p<0.01 and ***p<0.001; multiple comparisons: #p<0.05, ##p<0.01 and ###p<0.001). Control condition corresponds to non-irradiated, non-treated mice.

To validate *in vivo* the ability of NAC to abolish resistance of SCC to PDT induced by CAF‑derived TGFβ1, we photogenerated cSCC in SKH-1 mice by chronic exposition to UV light (Figure 5A). UV irradiation stopped and topical treatment with NAC started at the time point that 50% of the mice presented visible skin lesions (day 0) and was maintained until the end of the experiment (day 42). Two sessions of PDT were applied (at day 14 and day 27) and skin samples were collected at the end point. The timeline of the experiment is illustrated in Figure 5A.

The number of lesions was homogeneous in all mice at the time that UV irradiation ended (day 0). While no significant differences were detected in subsequent intermediate time points, the number of lesions was significantly reduced in mice belonging to the UV+NAC+PDT group compared to the rest of groups at the end point (day 42) (Figure 5B, S8A). To more accurately evaluate the efficacy of the treatment, we estimated the tumor burden (TB) of each experimental group by calculating the sum of the volumes of each tumor found on the mice belonging to that group. TB was then normalized by dividing the value by the number of mice (NM) composing the group at that specific time point. This analysis revealed that TB/NM was significantly lower in UV+NAC+PDT mice compared to other groups at day 42 (Figure 5C). In addition, a detailed analysis of the tumor size distribution evidenced that the vast majority of tumors present in the UV+NAC+PDT group belonged to the smallest range of size (<20 mm^3^) and, importantly, this was the only group lacking tumors over 100 mm^3^ volume at the end point (Figure 5D).

Histopathological analyses were also conducted in the skin samples collected. Epidermal thickness was significantly increased in UV-irradiated compared to non-irradiated control mice. Although single treatment with either PDT or NAC significantly reduced the epidermal thickening in response to chronic UV exposure compared to UV-irradiated non-treated mice, the combination of NAC+PDT was significantly more efficient, reaching similar values to those of the non-irradiated control (Figure 5E, 5F). In the same direction, the expression of the PCNA proliferation marker was significantly increased in the UV-irradiated non-treated mice compared to all UV-irradiated treated groups, but differentially decreased in UV-irradiated skin treated with NAC+PDT (Figure 5E, 5F). The expression of P53, a hallmark of UV-derived cellular damage, was also significantly decreased as a result of the combined treatment with NAC+PDT (Figure 5E, 5F).

In addition, TGFβ1 levels studied by ELISA in the blood samples longitudinally collected along the experiment strongly suggested the implication of TGFβ1 as a key target to restrain tumor growth in mouse skin, according to the significant drop in blood TGFβ1 levels resulting from the combined treatment of UV‑irradiated mice with NAC+PDT Figure S8B).

## 4. Discussion

The use of PDT for the treatment of cSCC represents a major advance in skin cancer therapeutics due to the high selectivity and the cosmetic outcome improvement of this approach. However, PDT relapse rate of five years after treatment is 18-50%, and up to 50% of the cases remain unresponsive (25-27. Many studies have pointed at TGFβ1 in the tumor microenvironment as one of the possible culprits of resistance to different therapies in various types of cancer 6. The design of new strategies to improve PDT including the combination of this clinical approach with compounds that enhance its action and avoid potential resistance is a major challenge. In this context, drug repurposing can be an excellent option. Based on previous data demonstrating the ability of NAC and raloxifene to significantly reduce TGFβ1 levels in human fibroblast cultures 19, our study proposes the use of NAC and raloxifene as co-adjuvants to increase the efficacy of PDT.

With the aim of recreating *in vitro* the TGFβ1-enriched environment driven by CAFs in cSCC, we exposed A431 and SCC13 cells to human CAF‑derived CM prior to PDT. CAF-derived conditioned medium induced resistance to PDT in A431 cells. Interestingly, when CAFs were treated with NAC or raloxifene before CM collection, the PDT resistance induced in A431 cells by T205A CAF‑derived CM was prevented. Given that increased TGFβ1 secretion by CAFs has been reported as a driver of resistance to PDT in A431 cells 23, our results are in agreement with the role of NAC and raloxifene as restrainers of TGFβ1 secretion by CAFs, which has been demonstrated with further experiments in this study. Thus, our work establishes for the first time NAC and raxolifene as PDT co-adjuvants to prevent therapy resistance. Importantly, spheroids constitute a powerful experimental system to more accurately mimic *in vivo* events related to tumor biology 28. The results obtained using this culture model not only support the enhanced efficacy of PDT when combined with NAC or raloxifene, but also evidence the ability of these compounds to limit the migratory capacity of PDT-treated A431 cells from spheroids 29,30.

Unlike raloxifene, NAC is a well-established antioxidant widely recognized for its role in ROS scavenging. It exerts this effect by modulating redox signaling within cancer cells. Notably, previous studies have demonstrated that NAC may directly react with ROS following NAC metabolization. In addition, it can increase glutathione levels, thereby enhancing ROS detoxification 31. Given that the efficacy of MAL‑PDT could be potentially compromised by this antioxidant effect, we confirmed that the use of NAC at the selected concentrations did not interfere. Specifically, the treatment of A431 cells with 0.1 mM NAC did not compromise their response to MAL‑PDT, demonstrating that NAC at this concentration does not impair MAL-PDT effectiveness. In fact, previous studies utilizing NAC as a ROS inhibitor employed substantially higher concentrations, approximately 10 mM NAC 32. In this scenario, our findings suggest that the effects observed in response to low concentrations of NAC may be more closely associated with the modulation of TGFβ1 signaling.

TGFβ1 biology comprises complex functions that are often cell type‑ and context-dependent. Increasing evidence suggests that the activation of the TGFβ1/SMAD signaling cascade in tumor cells promotes cell-cycle arrest, leading to the entry of tumor-propagating cancer cells into a quiescent state with decreased susceptibility to suffer cellular damage in response to therapeutic agents. In other cancer cell types, this effect has been related to the upregulation of P21 expression as a result of the nuclear translocation of the P‑SMAD2/3-P-SMAD4 complex 12. Given that P21 is a well-known mediator of P53-induced G1/S cell cycle arrest 10, P21 upregulation has been associated to resistance to other therapies such as cisplatin and doxorubicin 33,34. In this study, we report increased P‑SMAD2 and P21 levels in A431 cells in response to CAF-derived CM, which was prevented when T205A CAFs were treated with NAC and raloxifene prior to CM collection. In addition, the results of EdU incorporation assays suggested that CAF-derived CM differentially slowed down the G1/S transition in A431 cells, and this effect was partially restored when CM was obtained from NAC or raloxifene-treated T205A CAF cultures. These findings support the hypothesis that the use of NAC and raloxifene in this context is contributing to restore a more actively cycling state in cSCC cells and to limit the activity of the TGFβ1/SMAD signaling pathway, overall facilitating higher sensitivity to MAL-PDT. Noteworthy, the fact that no differences in P-SMAD and P21 levels or EdU incorporation rates were observed in SCC13 cells in response to CAF-derived CM is in agreement with the lack of CAF-derived CM-induced resistance to PDT in this cSCC cell line. These findings align with our previous research, where the lack of CAF-derived CM-induced resistance to PDT in SCC13 cells was attributed to the relatively high endogenous TGFβ1 production by this cSCC cell line 23.

NAC and raloxifene have previously been repurposed for the treatment of other cancer-related pathologies such as breast and pancreatic cancer. Both substances contribute to stimulate CAF endoglin expression and therefore negatively modulate the TGFβ1/SMAD2/3 pathway in cancer cells by decreasing extracellular TGFβ1 levels 19. Although endoglin upregulation in CAFs could be considered controversial as high levels of this TGFβ1 co-receptor have been associated to more aggressive cancer variants 35, here we provide evidence for the first time that the response of cSCC to PDT can be improved by using this strategy.

The data presented herein are in line with the ability of NAC and raloxifene to limit TGFβ1 production by T205A CAFs, which can be associated with increased levels of both membrane and soluble endoglin and ultimately prevent the development of resistance to PDT in A431 cells exposed to T205A‑derived CM. On the other hand, unlike T205A, T165A CAFs did not show differential levels of TGFβ1 secretion or TGFβ/SMAD pathway activation in response to NAC or raloxifene. This is in agreement with the persistence of PDT resistance in A431 cells exposed to T165A-derived CM, even when this CAF model was exposed to NAC or raloxifene. The differential response observed using these two different CAF cultures could be associated to the relatively higher membrane and soluble endoglin levels detected even in basal conditions in T205A compared to T165A. The regulation of the shedding process to produce soluble endoglin is another interesting feature to further investigate, as NAC and raloxifene modulate the levels of soluble endoglin in both T205A and T165 CAFs, but membrane endoglin levels remain unchanged in response to these compounds in T165A. In spite of the increase of soluble endoglin levels in T165A CAF‑derived CM as a result of the treatment with NAC or raloxifene, the soluble endoglin levels reached were similar to those obtained in basal conditions from T205A CAFs, which may explain the inability to revert resistance to PDT in A431 cells when using T165A CM. Finally, future studies will also need to address the implications of the *in situ* or invasive features of the tumor from which the CAF cultures are originated. In this work, increased endoglin levels are associated with lower TGFβ1 levels, pointing at endoglin as a key molecular mediator to prevent the resistance to PDT in cSCC cells. The exposition of T205A to recombinant endoglin led to decreased TGFβ1 secretion, which supported the role of endoglin in the modulation of the TGFβ1 pathway. Importantly, endoglin silencing in T205A CAFs was sufficient to block the response to NAC, as A431 cells exposed to CM from endoglin-silenced T205A CAFs treated with NAC remained resistant to PDT. Altogether, these results highlight endoglin as an essential element to tackle PDT resistance in A431 cells, and thus, a potential therapeutic target for cSCC treatment.

These findings are consistent with our previous research 23 demonstrating that the resistance to MAL‑PDT observed in A431 cells was attributed to the TGFβ1 secreted by CAFs. Notably, this resistance was abolished by using the SB525334 TGFβ1 receptor inhibitor to block TGFβ1 signaling, while PDT toxicity was reduced by incubating A431 cells with recombinant TGFβ1. Overall, these results collectively support the role of this cytokine in promoting resistance to MAL-PDT. Consequently, in the present work, we report improved effectiveness of MAL-PDT as a consequence of reducing TGFβ1 levels in CAF-derived CM by pre‑treating the CAFs with NAC or raloxifene to increase CAF endoglin levels.

Resistant lesions may originate from various sources. In this study, we model a situation in which resistance arises prior to treatment due to features of the tumor microenvironment, specifically high levels of TGFβ1 secreted by CAFs. In this sense, this study together with our previous work have demonstrated that this cytokine mediates resistance by inducing cell cycle arrest 23. Consequently, pre‑treating the tumor with anti-TGFβ1 agents could potentially prevent this cell cycle arrest effect before applying MAL-PDT, as a strategy to overcome resistance. In line with this, boosting endoglin production by CAFs can be proposed as a therapeutic tool to reduce the levels of secreted TGFβ1.

On the other hand, intrinsic features of cancer cells can also contribute to develop resistance to MAL-PDT. Related to this, compared to A431 cSCC cells, we have demonstrated that SCC13 cells secret higher levels of TGFβ1 and express lower levels of HO-1, correlating with lower basal ROS levels and lower basal sensitivity to PDT, which can be understood as intrinsic resistance to the treatment. In spite of this, the higher TGFβ1 basal levels in SCC13 prevent the development of additional resistance to PDT when exposed to a TGFβ1 enriched microenvironment, possibly due to autocrine signaling saturation. Conversely, A431 cells, with lower TGFβ1 secretion and basal HO-1 expression, show greater sensitivity to PDT when isolated but are more susceptible to develop resistance when exposed to exogenous TGFβ1, a response that is likely mediated by the induction of cell cycle arrest. These findings suggest that the intrinsic resistance of SCC13 cells to PDT is tied to the modulation of the heme synthesis pathway by its TGFβ1 basal levels rather than induced by TGFβ1 secreted by CAFs, whereas the resistance observed in A431 cells is linked to CAF-derived TGFβ1-mediated modulation of cell proliferation 23.

Alternatively, resistance to treatment can be developed as a result of the application of a series of multiple MAL-PDT sessions. In agreement with this, multiple doses of MAL-PDT appear to have a paradoxical effect: while they can be more effective than a single dose, they can also promote the selection and emergence of resistant cells. This effect has been observed clinically, as initially well-responding patients to multiple MAL-PDT sessions developed recurrent, more aggressive lesions, suggesting that PDT in combination with other factors may contribute to the selection of highly aggressive tumor cells 36,37. This phenomenon has also been reproduced by our group using *in vitro* models to obtain resistant cells after MAL-PDT treatment by isolating surviving cells and subjecting them to further MAL-PDT sessions, as a tool to identify distinctive features of resistant cells 38. Thus, characterizing the lesion prior to MAL-PDT application is a crucial step to anticipate the potential outcome and select the most appropriate treatment.

Finally, to validate the abolishment of resistance to PDT in cSCC, we used the SKH-1 immunocompetent mouse model that selectively develops SCC when subjected to skin photocarcinogenesis by chronic irradiation with UV 39-40. Compared to PDT, the combination of PDT with NAC differentially limited tumor development and growth, especially in the long term, as major differences were obtained at the endpoint of the experiment. This outcome could be associated to the decrease of the TGFβ1 levels in the circulation of mice treated with the combination of PDT+NAC. Finally, the reduction in the epidermal levels of PCNA and P53 as a result of the combined treatment with NAC+PDT was indicative of reduced UV-induced damage. Overall, these findings place NAC as a powerful co-adjuvant tool to enhance the effectiveness of MAL-PDT treatment for cSCC by preventing the development of resistance.

These findings hold significant potential for advancing personalized anti-cancer therapies in clinical settings. Specifically, assessing the levels of biomarkers related with TGFβ1 signaling and cellular quiescence —such as endoglin, P-SMAD2 and P21— in tumor biopsies, alongside measuring TGFβ1 levels in patient serum, could provide valuable insights to anticipate the appearance of resistant responses to MAL-PDT. This approach would motivate the development of tailored therapeutic strategies to overcome these difficulties and optimize treatment outcomes for individual patients.

## 5. Conclusions

In conclusion, our study shows that CAF-derived CM can promote resistance to PDT by inducing quiescence in cSCC cells as a result of activated TGFβ1 signaling. Our data highlight the ability of NAC and raloxifene to enhance endoglin expression in CAFs as a potent tool to overcome TGFβ1-induced resistance to PDT in cSCC cells. In addition, our findings suggest that the effectiveness of PDT may depend on cell‑autonomous features of cSCC cells that differ among distinct research models and patients. Overall, our work emphasizes the potential of targeting endoglin expression in CAFs to optimize PDT, providing novel insights towards more personalized treatment approaches for cSCC tumors.

## Supplementary Material

Supplementary figures.

## Abbreviations

CAF: Cancer-associated fibroblast
CM: Conditioned medium
cSCC: Cutaneous squamous cell carcinoma
DMEM: Dulbecco's Modified Eagle Medium
Fb: Control fibroblast
FBS: Fetal bovine serum
MAL: Methyl-aminolevulinate
NAC: N-acetyl cysteine
PDT: Photodynamic therapy
PI/OA: Propidium iodide/orange acridine
TGFβ1: Transforming growth factor β1
UV: Ultraviolet
