# Supplementary material for: N-acetylcysteine and raloxifene boost photodynamic therapy against cutaneous squamous cell carcinoma by decreasing TGFβ1 secreted by cancer-associated fibroblasts
Source: Int J Biol Sci. 2025 Apr 28;21(7):3164–82. doi: 10.7150/ijbs.106642 (PMC12080389; doi:10.7150/ijbs.106642)
Supplement: Supplementary file 1 — Supplementary figures. [file ijbsv21p3164s1.zip › legends.pdf]

Figure S1. Effect of the treatment with NAC or raloxifene on control fibroblasts (Fb), and on A431 cells in the presence or absence of Fb-derived CM. (A) Upper panel: Cell viability rates by MTT of Fb, A431 cells and A431 cells co-cultured with Fb-derived CM in the presence of different concentrations of NAC and raloxifene. Lower panel: Phase contrast images illustrating the morphological changes in the morphology directly induced by the incubation of each cell type with NAC or raloxifene. Scale bar: 50 µm. Data were normalized to untreated cells. Error bars denote ± S.E.M. (n = 3, one-way ANOVA: statistical comparison to only MAL condition: *<0.05, **p<0.01 and ***p<0.001). (B) Upper panel: Cell viability rates by MTT of A431 cells treated with MAL-PDT (0.5 mM of MAL and red light dose of 6.1 and 9.1 J·cm^{-2}) in the presence or absence of Fb-derived CM and selected concentrations of NAC and raloxifene. Lower panel: Phase contrast images illustrating the morphological changes in the morphology directly induced by the incubation with each treatment and MAL-PDT (0.5 mM of MAL and red light dose of 6.1 J·cm^{-2}). Scale bar: 50 µm. Error bars denote ± S.E.M. (n = 3, one-way ANOVA: statistical comparison to only MAL condition: *<0.05, **p<0.01 and ***p<0.001).

Figure S2. Comparative analysis of the response to PDT of cSCC cultures exposed to different treatment conditions. (A) Upper panel: phase contrast images illustrating the morphological changes in the morphology of bidimensional cSCC cultures directly induced by the incubation with CAF-derived CM. Scale bar: 50 µm. Lower panel: cell viability rates of A431 and SCC13 cells treated with MAL-PDT (0.5 mM of MAL and red light dose of 6.1 J·cm^{-2}) in the presence of each type of CM. (B) Differential PPIX accumulation in cSCC cell lines exposed to increasing concentrations of TGFβ1. Individual dots in the graphs represent technical replicates of each experimental condition. (C) Left: representative fluorescence microscopy images of A431 spheroids. Alive cells are visualized in green under blue light irradiation (450-490 nm); and dead cells in red under green light irradiation (570-590 nm). Scale bar: 200 µm. Right: cell viability and size of A431 spheroids in response to MAL-PDT (0.5 mM of MAL, red light dose of 6.1 J·cm^{-2}) in the presence of CM from Fb and T205A, pre-treated or not with NAC or raloxifene. Cell viability was evaluated by PI/OA assay. Size was estimated by measuring the diameter of the spheroid in the images taken 24 h after MAL-PDT. Error bars denote ± S.E.M. (n = 3, one-way ANOVA, statistical comparisons to only MAL condition: *<0.05, **p<0.01 and ***p<0.001; multiple comparisons: #p<0.05, ##p<0.01 and ###p<0.001).

Figure S3. Effect of CM from CAFs in the presence or absence of NAC or raloxifene on the TGFβ1/SMAD2/3 pathway in SCC13 cells. (A) Localization of SMAD2/3 analyzed by IF. Hoechst-33258 (Ho) was used for nuclear counterstaining. Scale bar: 20 µm. (B) P-SMAD 2 and SMAD2 analysis by Western blot in SCC13 cells. Bands between the dotted lines (lanes 2 and 3 for the NAC assay and lanes 1, 2 and 4 for the raloxifene assay) correspond to protein extracts of no interest for the present work. Western blot images are representative of n = 3 independent experiments. (C) Analysis of the expression of P-SMAD2/SMAD2 ratio in SCC13 cells using ImageLab software. All the results are relativized to Fb-derived CM treatment. Error bars denote ± S.E.M. (n = 3, one-way ANOVA, statistical comparisons to Fb condition: *<0.05, **p<0.01 and ***p<0.001; multiple comparisons: #p<0.05, ##p<0.01 and ###p<0.001). (D) EdU labelling detection in SCC13 cells using fluorescence microscopy. Upper row: fluorescence microscopy images revealing EdU incorporation. Lower row: nuclear counterstaining with Hoechst-33258. All images were taken at the same magnification. Scale bar: 50 µm. (E) Quantification of the percentage of EdU positive nuclei in SCC13 cells. All the results are relativized to Fb-derived CM treatment. Error bars denote ± S.E.M. (n = 3, one-way ANOVA, statistical comparisons to Fb condition: *<0.05, **p<0.01 and ***p<0.001; multiple comparisons: #p<0.05, ##p<0.01 and ###p<0.001). (F) Expression of P21 analyzed by Western blot in SCC13 cells. Bands between the dotted lines (lanes 2 and 3) correspond to protein extracts of no

interest for the present work. Western blot images are representative of n = 3 independent experiments. (G) Analysis of the expression of P21 in SCC13 cells using ImageLab software. All the results are relativized to Fb-derived CM treatment. Error bars denote ± S.E.M. (n = 3, one-way ANOVA, statistical comparisons to Fb condition: *<0.05, **p<0.01 and ***p<0.001; multiple comparisons: #p<0.05, ##p<0.01 and ###p<0.001).

Figure S4. Full Western blot membranes from Figure 2 in the main text.

Figure S5. Full Western blot membranes from Figure S3 in supplementary material.

Figure S6. Full Western blot membranes from Figure 3 in the main text.

Figure S7. Full Western blot membranes from Figure 4 in the main text.

Figure S8. *In vivo* assay combining NAC+PDT treatment in SKH-1 mice chronically exposed to UV light. (A) Representative images of the back skin of the mice at day 14 (prior to the first PDT session) and at day 42 (end point). (B) Analysis of TGFβ1 levels in mouse serum (n = 4 for UV + PDT and UV + NAC group, n = 5 for UV + NAC + PDT group; one-way ANOVA: statistical comparison to UV condition: *p<0.05, **p<0.01 and ***p<0.001; multiple comparisons: #p<0.05, ##p<0.01 and ###p<0.001).
