# Supplementary figures and images for: N-acetylcysteine and raloxifene boost photodynamic therapy against cutaneous squamous cell carcinoma by decreasing TGFβ1 secreted by cancer-associated fibroblasts
Source: Int J Biol Sci. 2025 Apr 28;21(7):3164–82. doi: 10.7150/ijbs.106642 (PMC12080389; doi:10.7150/ijbs.106642)

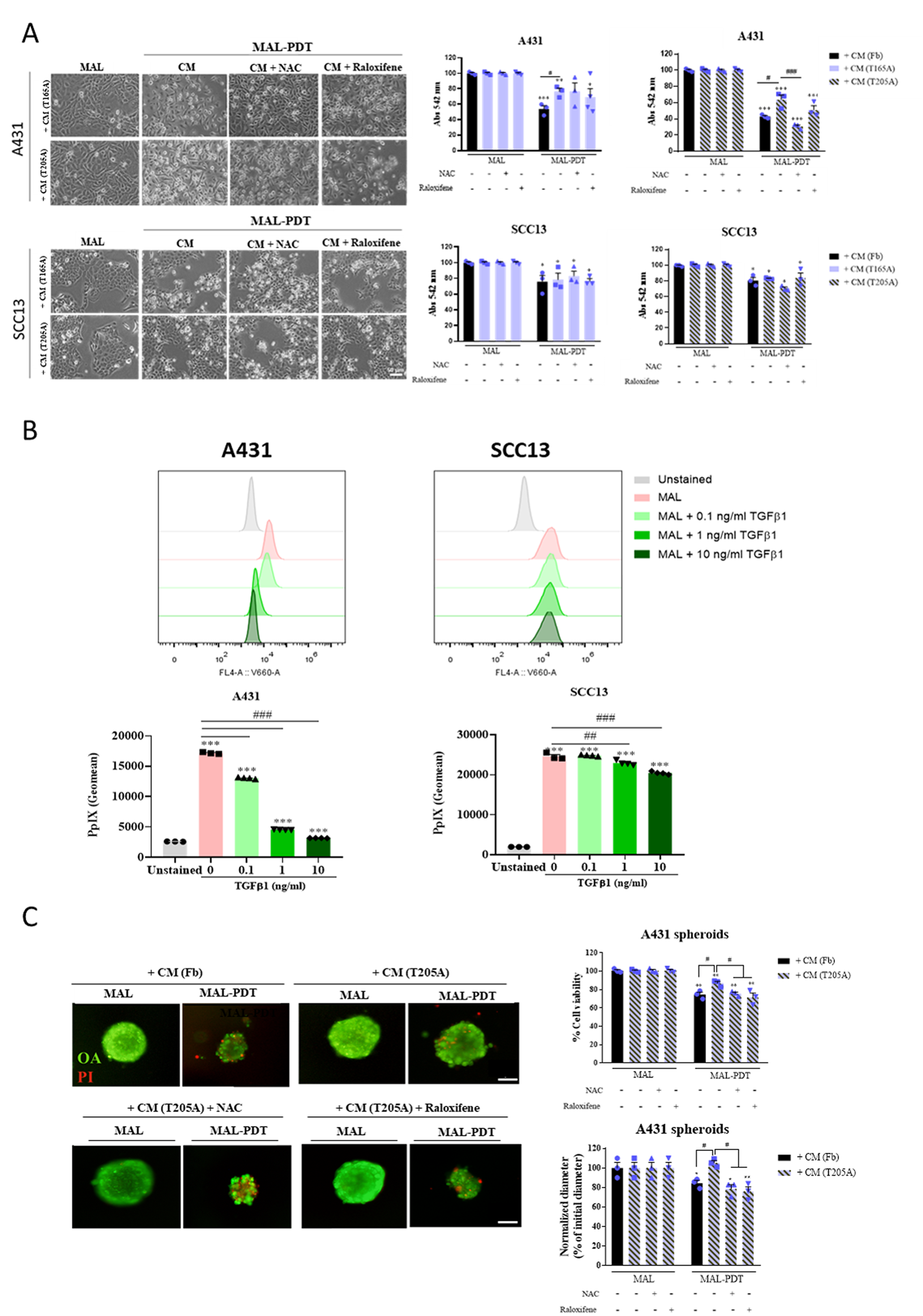

Supplement: Supplementary file 1 — Supplementary figures. [file ijbsv21p3164s1.zip › Figure S2.png]

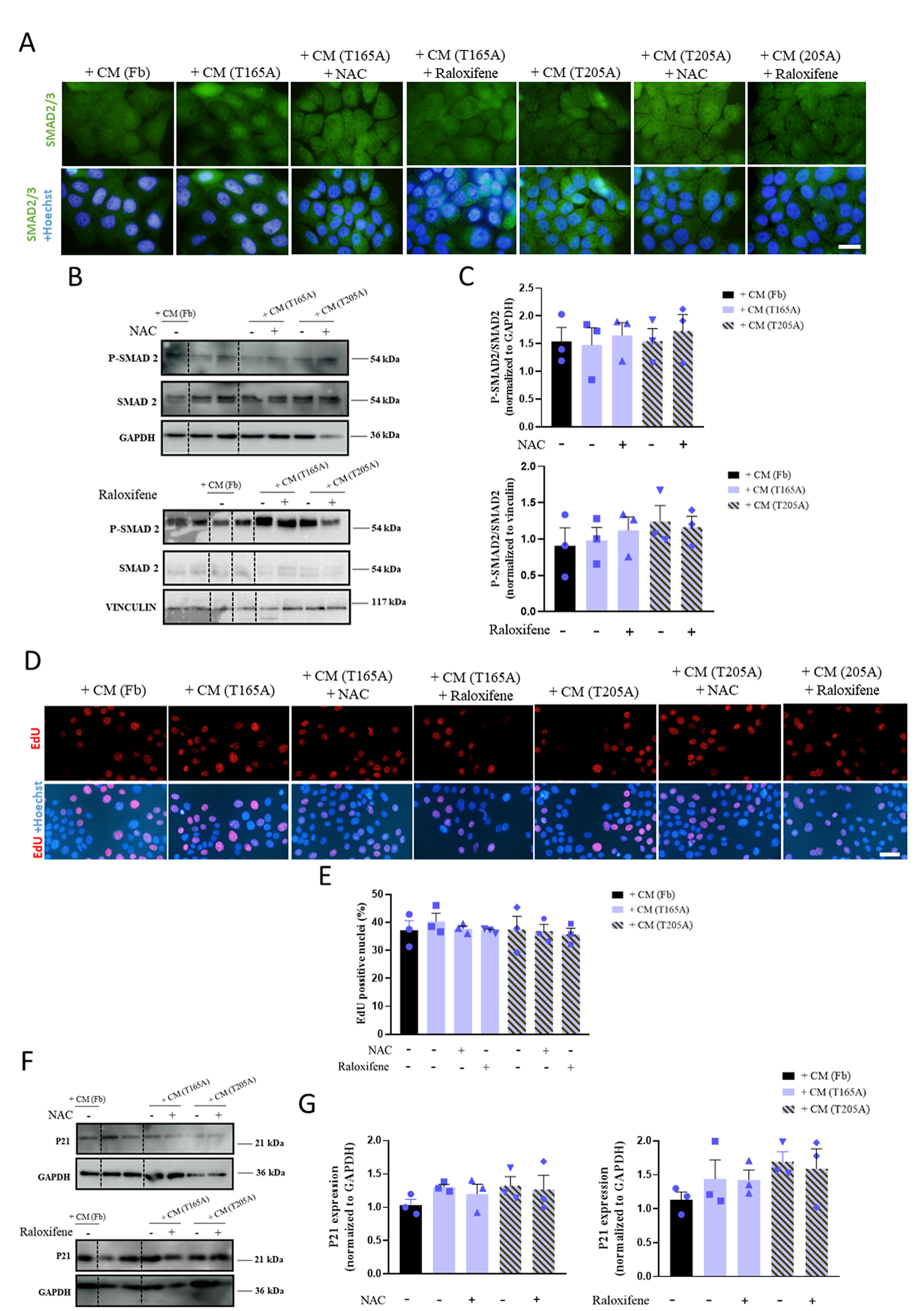

Supplement: Supplementary file 1 — Supplementary figures. [file ijbsv21p3164s1.zip › Figure S3.png]

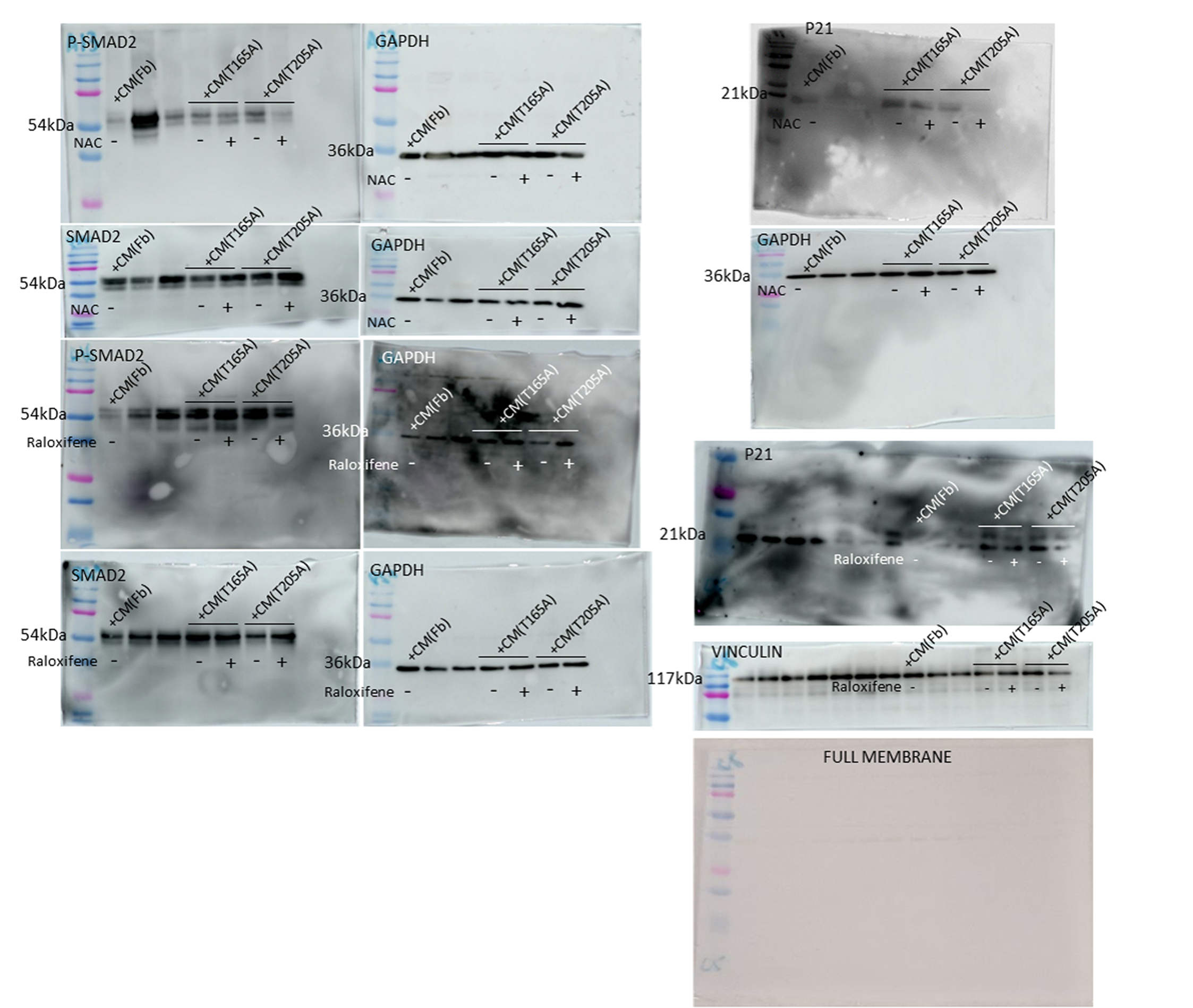

Supplement: Supplementary file 1 — Supplementary figures. [file ijbsv21p3164s1.zip › Figure S4.png]

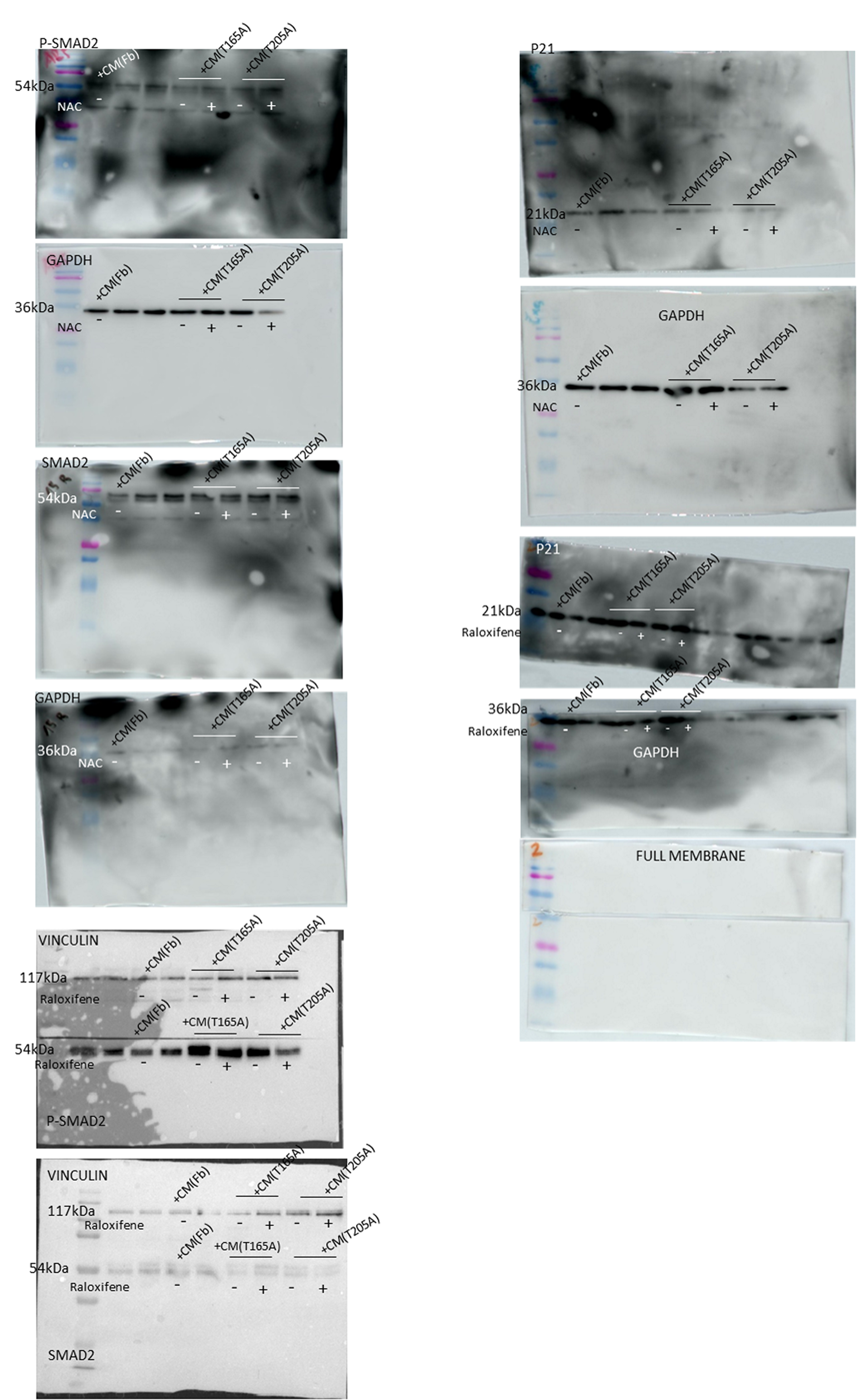

Supplement: Supplementary file 1 — Supplementary figures. [file ijbsv21p3164s1.zip › Figure S5.png]

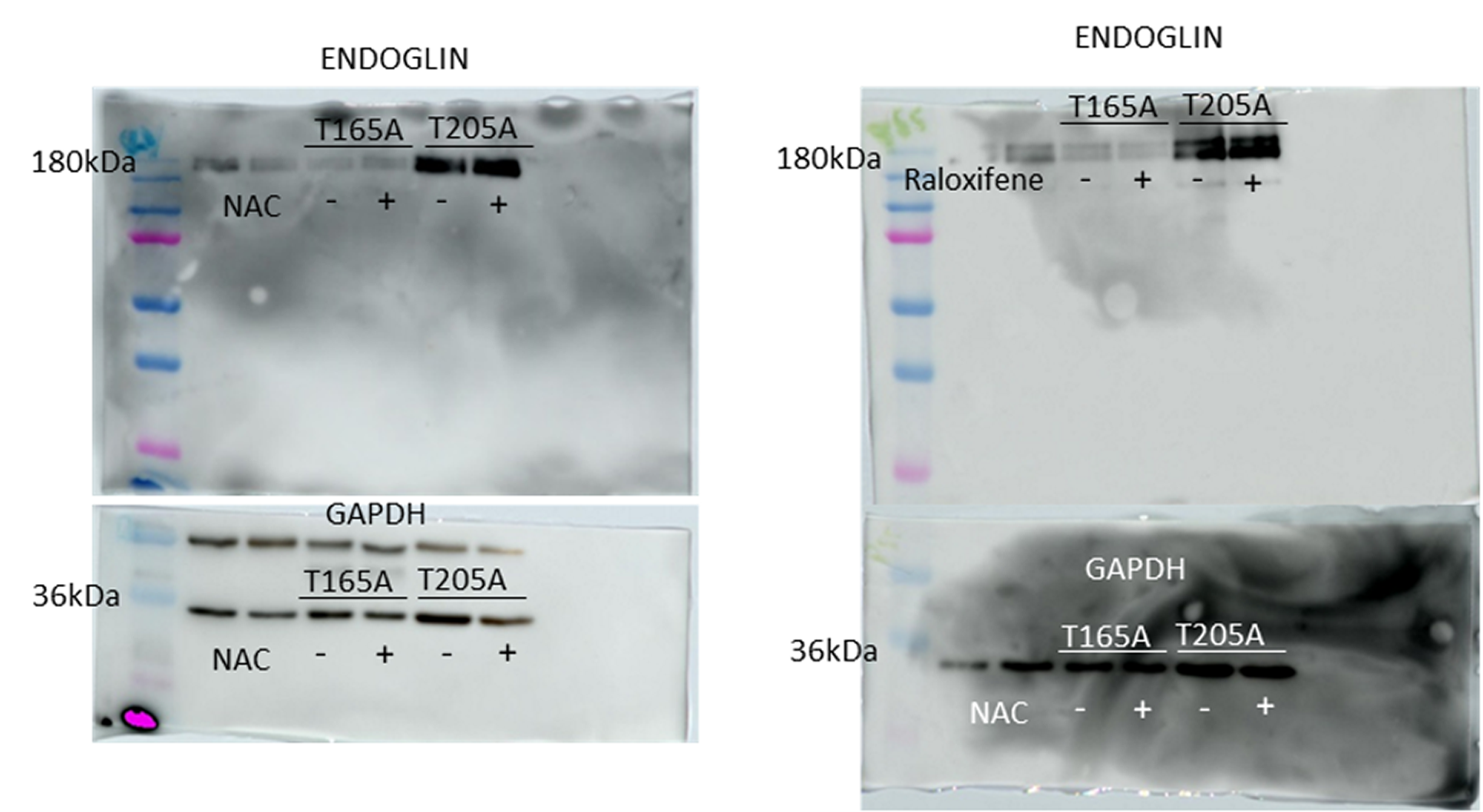

Supplement: Supplementary file 1 — Supplementary figures. [file ijbsv21p3164s1.zip › Figure S6.png]

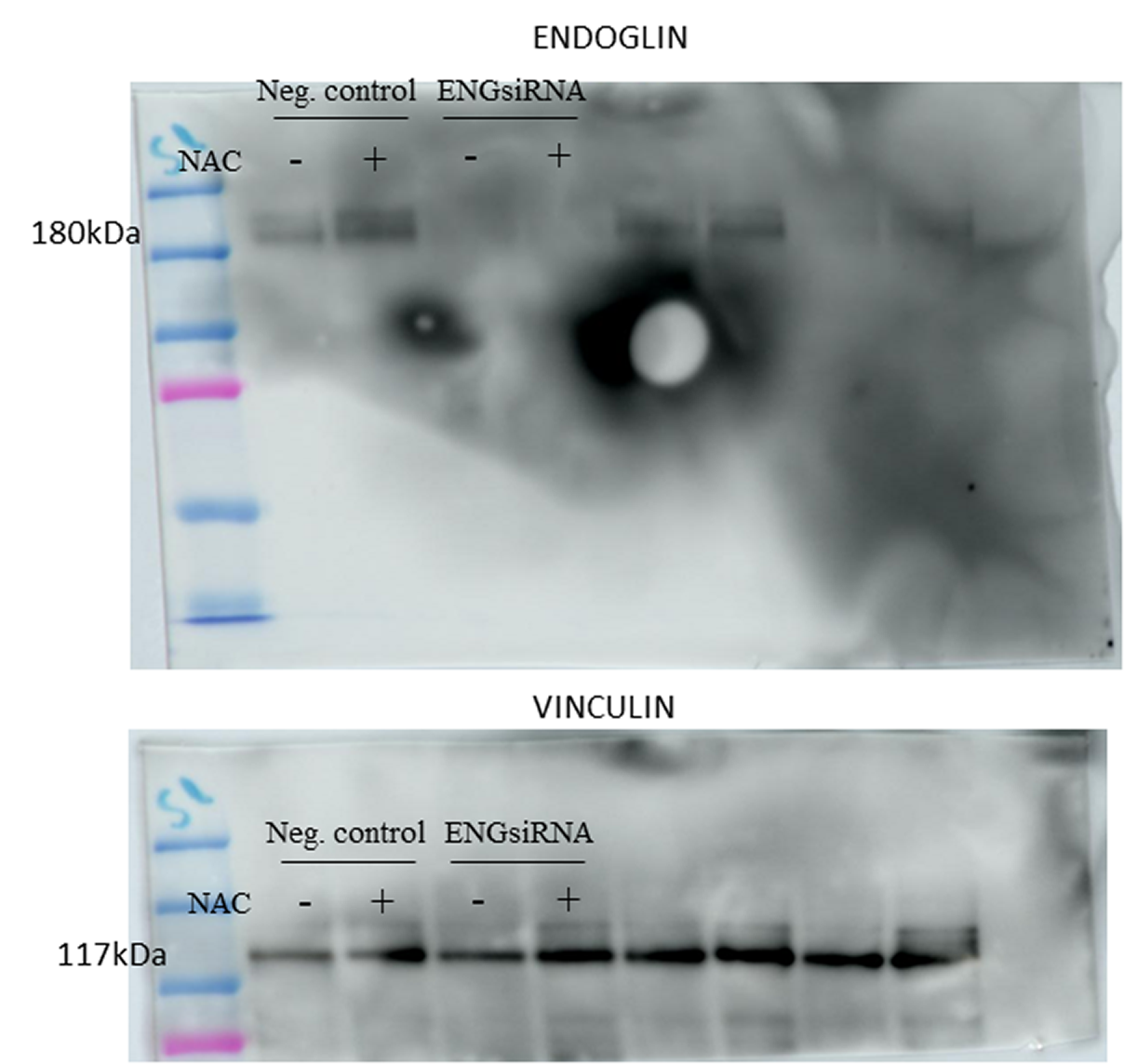

Supplement: Supplementary file 1 — Supplementary figures. [file ijbsv21p3164s1.zip › Figure S7.png]

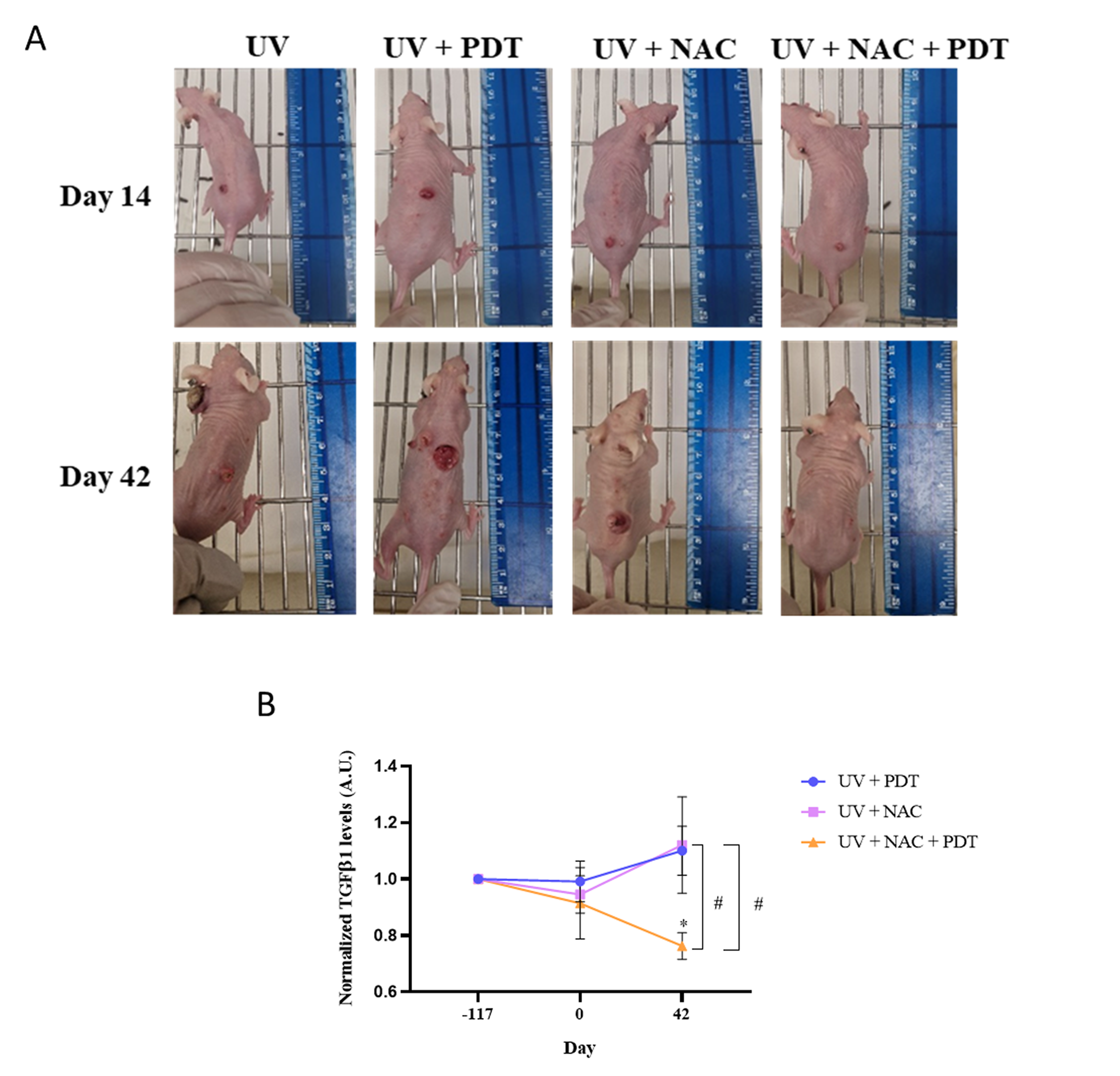

Supplement: Supplementary file 1 — Supplementary figures. [file ijbsv21p3164s1.zip › Figure S8.png]

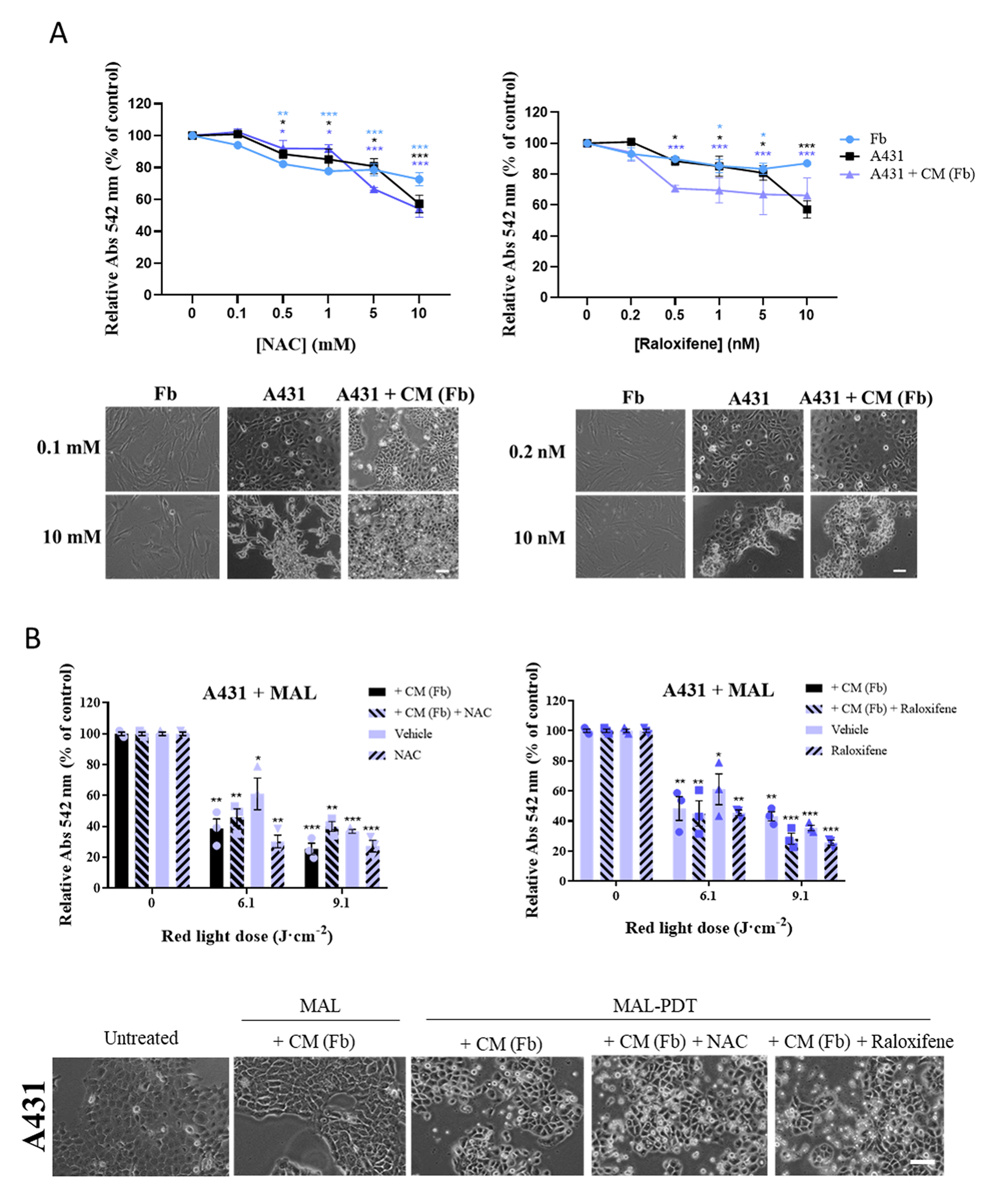

Supplement: Supplementary file 1 — Supplementary figures. [file ijbsv21p3164s1.zip › Figure S1.png]
